# CS-[BiC-PiC]-NiCl_4_: a green and effective heterogeneous catalyst for one-pot synthesis of 2,3-dihydroquinazolin-4(1*H*)-one derivatives

**DOI:** 10.1039/d5ra07849k

**Published:** 2026-02-11

**Authors:** Narges Seyedi, Maryam Mousapour, Mohammad Rizehbandi, Farhad Shirini

**Affiliations:** a Department of Organic Chemistry, Faculty of Chemistry, University of Guilan Rasht 41335-19141 Iran shirini@guilan.ac.ir +98 131 3233262 +98 131 3233262

## Abstract

In this study, a novel nickel ion-containing dicationic ionic liquid immobilized on chitosan (CS-[BiC-PiC]-NiCl_4_) was designed, synthesized, and fully characterized by using various techniques such as Fourier transform infrared spectroscopy (FT-IR), ^1^H NMR and ^13^CNMR spectra, X-ray diffraction (XRD), thermogravimetric analysis (TGA), field emission scanning electron microscopy (FESEM), energy dispersive X-ray spectroscopy (EDS), and inductively coupled plasma (ICP) analysis. The theoretical electronic structure, catalytic mechanism, and identity of most of the active sites in the CS-[BiC-PiC]-NiCl_4_ system were investigated using a suite of computational methods, including density functional theory (DFT), molecular electrostatic potential (MEP) mapping, quantum theory of atoms in molecules (QTAIM), and reduced density gradient (RDG) analysis. Its potential was demonstrated in the facile and eco-friendly synthesis of pharmacologically important 2,3-dihydroquinazolin-4(1*H*)-one derivatives using two distinct protocols, including the reaction of 2-aminobenzamide with aldehydes and the reaction of isatoic anhydride with ammonium acetate and aldehydes under neat conditions at 120 °C, demonstrating excellent recyclability over five runs, achieving high yields (93–97%) with short reaction times (3–8 minutes). This offers a sustainable and practical synthetic platform, facilitated by the synergistic interplay between the nickel Lewis acid sites and the chitosan support. This process provides significant advantages, including operational simplicity, solvent-free conditions, straightforward product isolation, and easy recovery of a cost-effective catalyst, which demonstrated exceptional stability over multiple cycles with no detectable nickel leaching, underscoring its truly heterogeneous nature. Consequently, this methodology represents a sustainable and practical approach for the synthesis of these valuable heterocycles.

## Abstract 2

In this study, CS-[BiC-PiC]-NiCl_4_ was designed, synthesized, and fully characterized by using various techniques. In continue this reagent was used for the promotion of the synthesis of 2,3-dihydroquinazolin-4(1*H*)-one derivatives.

## Graphical abstract

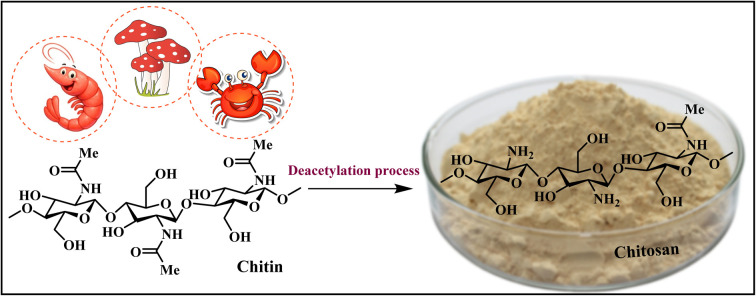

## Introduction

Catalysis is fundamental to most industrial chemical processes. Among these, heterogeneous or supported catalysts are generally preferred because they enable straightforward product separation and facilitate catalyst recovery for recycling. Recently, catalysts derived from bioresources have attracted significant interest due to their economic and environmental benefits. These materials are typically non-toxic, biodegradable, and inherently heterogeneous, allowing for easy recovery and reuse. Consequently, biopolymers like chitosan (CS) have become increasingly popular as support materials, aligning with the growing emphasis on sustainable and economically viable chemical processes.^1–4^

Beyond its favorable intrinsic properties, chitosan, as a value-added product derived from chitin (a primary component of crustacean shell), is the second most abundant biopolymer in the world and represents a prime example of a biomass-derived material. Its production aligns with the core principles of green chemistry by valorizing raw streams and converting biological resources into high-value functional polymers.^5,6^

**Fig. 1:**
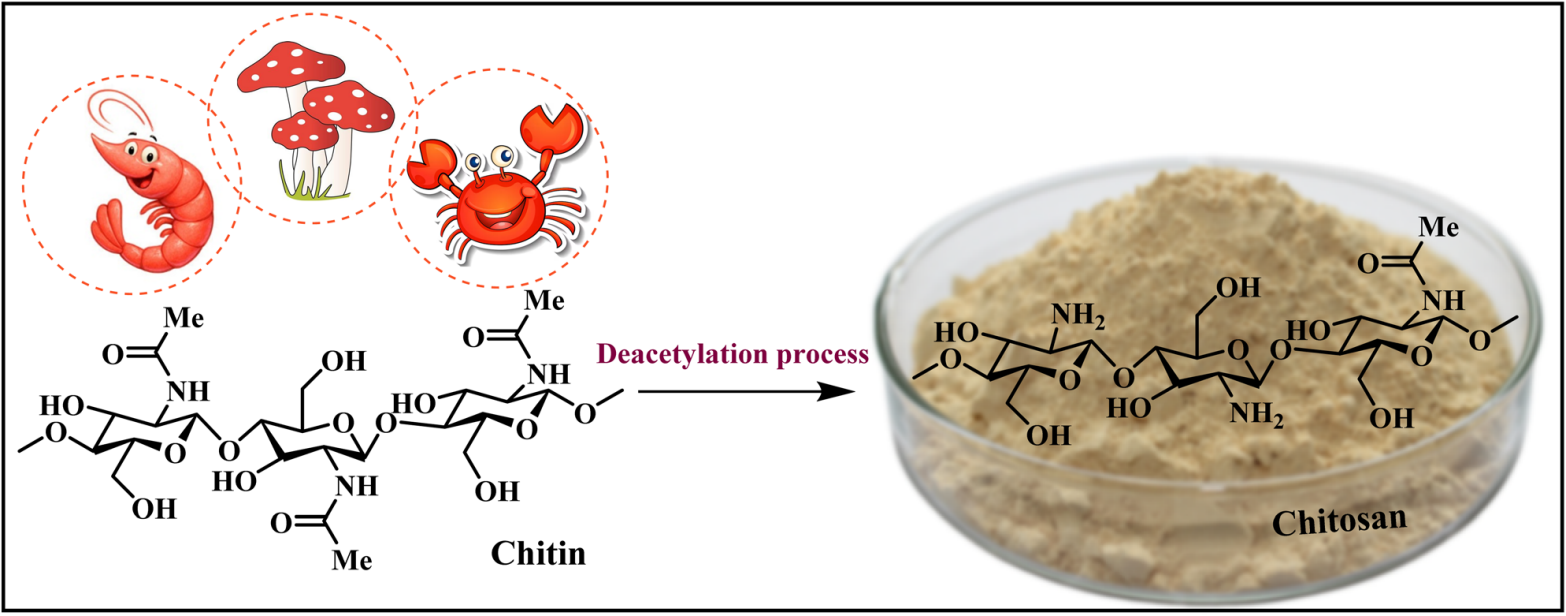
Structure of chitin and chitosan.

The complex molecular structure of chitosan (CS), which features amine and hydroxyl functional groups from its β-(1–4)-linked d-glucosamine and *N*-acetyl-d-glucosamine units (Fig. 1), enables its versatile application in fields such as drug delivery,^7^ biosorption,^8^ adhesives,^9^ membranes,^10^ and especially, catalysis.^11,12^ In particular, its reactive amino, and primary and secondary hydroxyl functional groups can be used to induce chemical modifications, either covalently or noncovalently. This makes the polymer an excellent solid support for surface modification with ionic liquids, leading to efficient heterogeneous catalysts for chemical transformations.^13,14^

Ionic liquids (ILs) represent one of the most rapidly growing areas of research in materials chemistry over the last decade. Conventionally, ILs are composed entirely of cations and anions and have a melting point below 100 °C.^15,16^ Consequently, they are typically liquid under ambient conditions. They are considered alternatives to hazardous and volatile organic compounds due to their unique physicochemical properties, such as negligible vapor pressure, non-flammability, high catalytic activity, and recyclability.^17,18^ Dicationic ionic liquids (DILs) are a novel and fascinating class of ionic liquids that contain two cationic moieties connected by a spacer, along with two anionic moieties. DILs have recently received increasing attention due to their distinct and appealing advantages compared with traditional monocationic ones, such as higher thermal and chemical stability, excellent structural tunability, superior heat capacity and so on.^19–21^

Despite their unique advantages, ionic liquids (ILs) present certain practical limitations. These include high viscosity, which can impede substrate diffusion, and challenges in their separation and purification from the reaction mixture, whether employed as a catalyst or as a solvent. To mitigate these issues and improve economic viability, the immobilization of ILs onto solid supports has emerged as a promising strategy. This approach has garnered significant global interest, as supported ionic liquids combine the beneficial properties of both the IL and the support, often yielding new, synergistic functionalities.^22–24^

As the fourth most abundant transition metal, nickel is a mainstay in heterogeneous catalysis due to its cost-effectiveness and natural prevalence, with a considerable portion of its global output dedicated to catalysts. Enhancing its performance typically involves creating nanostructures to maximize surface area. This design creates a powerful synergy between nickel's reactivity, the tunable properties of ionic liquids, and chitosan's biodegradable framework. The ionic liquid component acts as a molecular-level modulator, stabilizing the nickel species and electronically tailoring their active sites to boost selectivity.^25–27^

Furthermore, one of the environmentally friendly methods for the synthesis of organic compounds is the use of one-pot multicomponent reactions (MCRs). These reactions involve more than two components and are considered a powerful and efficient strategy for the synthesis of structurally complex and diverse organic molecules. The main advantages of MCRs are the reduction of the number of reaction steps and the minimized formation of by-products.^28,29^

For the past decade, the synthesis of heterocyclic compounds has drawn significant interest in academic and industrial settings due to their important role in fields such as drug discovery and development, medicinal and pharmaceutical research.^30^ Among the heterocycles, nitrogen-containing frameworks represent a highly valuable and significant class in organic chemistry.^31,32^

**Fig. 2:**
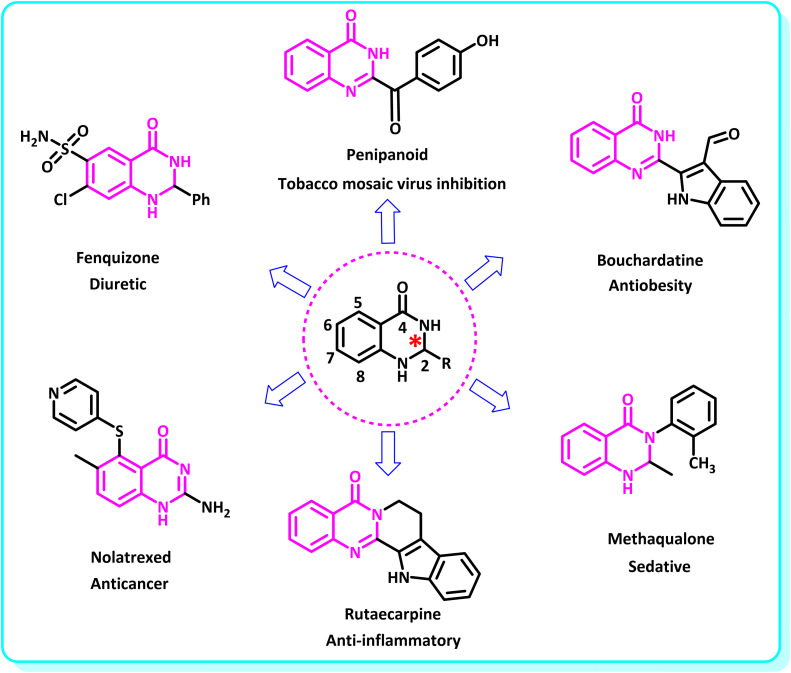
Some examples of bioactive compounds containing quinazolinone skeleton.

2,3-Dihydroquinazolin-4(1*H*)-one derivatives are an important class of nitrogen-containing heterocycles atom that exhibit a wide range of pharmacological and biological activities. penipanoid, bouchardatine, fenquizone, and methaqualone are representative drugs containing the quinazolinone core scaffold (Fig. 2). Numerous quinazolinone derivatives display a wide range of medicinal activities, including antibacterial,^33^ antiviral,^34^ antitumor,^35^ antihistamine,^36^ antiinflammatory,^37^ antifungal,^38^ anticonvulsant,^39^ antihypertensive,^40^ and anticancer^41^ properties, as well as potent human immunodeficiency virus (HIV) and other remarkable biological, pharmacological, and chemical effects. Currently, there is a significant interest in the synthesis of 2,3-dihydroquinazolin-4(1*H*)-one derivatives because of their ability to be readily oxidized to 2-substituted 4(3*H*)-quinazolinones. These quinazolinone derivatives are key building blocks in the creation of biologically active heterocyclic compounds.

Considering the importance of these compounds, a variety of heterogeneous catalysts such as montmorillonite-KSF clay,^42^ MCM-41@serine@Cu(ii),^43^ [hercynite@SiO_2_-l-arginine–Ni],^44^ nano [(Asp-Gua) IL@PEGSiO_2_] ^45^ and Fe_3_O_4_/SBA-15 (ref. 46) have been employed to accomplish their synthesis *via* MCR strategy using two distinct methods, including the reaction of 2-aminobenzamide with aldehydes and the three-component reaction of isatoic anhydride with ammonium acetate and aldehydes, under various conditions. Most of these methods have limitations, including long reaction times, the use of hazardous organic solvents, low yields, side reactions that produce mixtures of products, and tedious work-up procedures. Therefore, there is still a demand for the development of environmentally benign, simple, and high-yielding protocols for the synthesis of these desirable compounds.

In continuation of our efforts in designing heterogeneous catalytic systems,^47,48^ we herein report a novel heterogeneous catalyst, comprising a nickel ion-containing dicationic ionic liquid immobilized on chitosan, for the simple and environmentally benign synthesis of 2,3-dihydroquinazolin-4(1*H*)-ones. A key innovative aspect of this catalyst is its multi-functional design, which strategically integrates a reusable ionic liquid with a biodegradable polymer support. This synergy fulfills several green chemistry objectives simultaneously, offering a recoverable system that significantly reduces the need for hazardous solvents and paves the way for more sustainable synthetic methodologies.

## Experimental

All solvents and chemicals used were analytical grade, sourced from Merck (Munich) and Sigma-Aldrich (Mumbai), and were utilized without additional purification. Reaction progress and substrate purity were monitored by thin-layer chromatography (TLC) on silica-gel polygram SILG/UV 254 plates. The structural identity of the products was confirmed through their physical constants, comparison with authentic samples, and analysis by FT-IR and NMR spectroscopy.

### Characterization techniques

The Fourier-transform infrared spectra (FT-IR) were acquired on a VERTEX 70 spectrometer (Brucker, Germany) instrument using the KBr pellet method across the 4000–400 cm^−1^ spectral range. The ^1^H and ^13^C NMR spectra were recorded using Bruker BioSpin GmbH (Germany) operating at, 400, 500 and 600 MHz, equipped with standard probes and software. Spectra were obtained in DMSO-*d*_6_ and CDCl_3_ using tetramethylsilane (TMS) as an internal standard. X-ray diffraction (XRD) analysis was conducted using an X'Pert Pro instrument (Panalytical Company, Netherlands). Field emission scanning electron microscopy (FESEM) was performed using a TE-SCAN model Sigma VP (ZEISS Company in Germany). Thermogravimetric analysis (TGA) was carried out on a METTLER TOLEDO TGA/SDTA 851e instrument (Swiss). The catalyst's metal content was quantified by inductively coupled plasma (ICP-OES) on a Thermo Scientific ESPECTRO ARCOS instrument.

### Synthesis of 1,4-bis(4-chlorobutyl)piperazine-1,4-diium chloride (BiC-PiC)

**Scheme 1:**
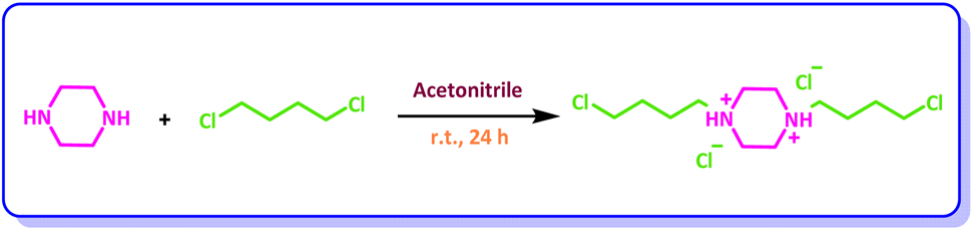
Preparation of 1,4-bis(4-chlorobutyl)piperazine-1,4-diium chloride.

A solution of piperazine (0.86 g, 10 mmol) in acetonitrile (30 mL) was prepared in a round-bottomed flask, and 1,4-dichlorobutane (2.2 mL, 20 mmol) was added to it. The reaction mixture was stirred at room temperature for 24 hours. The resulting precipitate was collected, washed with acetonitrile (2 × 10 mL), and dried at 70 °C to afford 1,4-bis(4-chlorobutyl)piperazine-1,4-diium chloride as a white solid (Scheme 1).^49^

### Synthesis of 1,4-bis(4-chlorobutyl)piperazine-1,4-diom nickel chloride (CS-[BiC-PiC]-NiCl_4_)

**Scheme 2:**
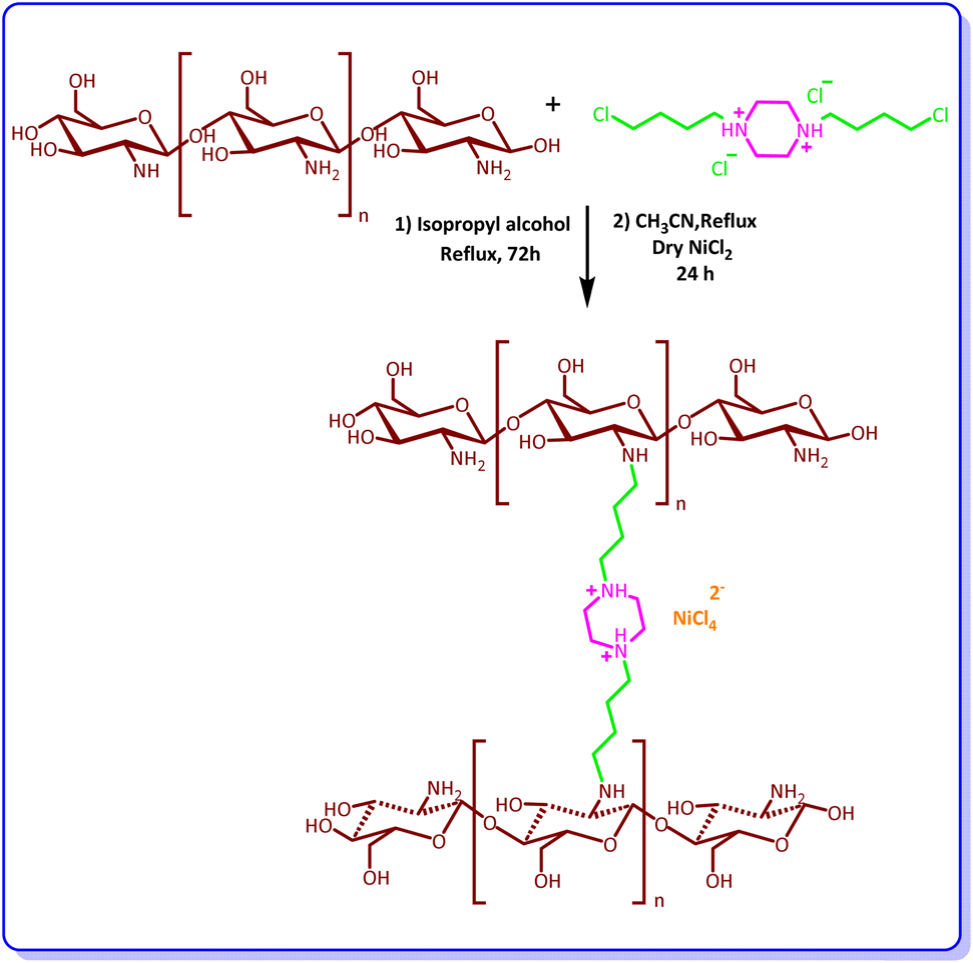
Preparation of 1,4-bis(4-chlorobutyl)piperazine-1,4-diium nickel tetrachloride (CS-[BiC-PiC]-NiCl_4_).

At this step, 3 mmol of the synthesized ionic liquid was added to 1 g of CS dispersed in isopropyl alcohol (30 mL). The reaction mixture was refluxed for 72 h, and then the obtained solid was washed with methanol to remove excess ionic liquid and dried at 70 °C. In the next step, nickel chloride (1 mmol) was added to the reaction product in acetonitrile solvent (30 mL) and the resulting mixture was stirred for 24 hours under reflux conditions. After filtering, the obtained product was washed with diethyl ether and then dried under vacuum conditions until the final product [1,4-bis(4-chlorobutyl)piperazine-1,4-diom nickel chloride on CS (1.929 g)] was obtained (Scheme 2).

### Synthesis of 2,3-dihydroquinazolin-4(1*H*)-ones (method A)

For the reaction, to a mixture of isatoic anhydride (1 mmol), aryl aldehyde (1 mmol), and ammonium acetate (2 mmol), CS-[BiC-PiC]-NiCl_4_ (20 mg) was added. Then the reaction mixture was stirred at 120 °C for the appropriate time. Upon completion, as indicated by TLC (*n*-hexane : ethyl acetate, 5 : 2), ethanol (10 mL) was added to the mixture, and the catalyst was separated by filtration. After evaporation of the solvent from the filtrate, the corresponding product was obtained with high purity in good to excellent yields.

### Synthesis of 2,3-dihydroquinazolin-4(1*H*)-ones (method B)

A mixture of 2-aminobenzamide (1 mmol) and aryl aldehyde (1 mmol), and CS-[BiC-PiC]-NiCl_4_ (20 mg) was stirred at 120 °C for the appropriate time. The progress of the reaction was monitored by TLC [*n*-hexane : ethyl acetate (5 : 2)]. After the completion of the reaction, ethanol (10 mL) was added to the mixture and the catalyst was separated by filtration. After evaporation of the solvent, the desired product was obtained with high purity in good to excellent yields.

### Catalyst recovery and reusability procedure

Reusability is a critical metric of a catalyst's alignment with green chemistry principles. To assess this, the model reaction between 4-chlorobenzaldehyde (1 mmol), isatoic anhydride (1 mmol), and ammonium acetate (2 mmol) was conducted under optimal conditions. Upon completion of the reaction (monitored by TLC, *n*-hexane : ethyl acetate, 5 : 2), ethanol (10 mL) was added to the mixture. The catalyst was then separated by filtration, washed, dried under vacuum, and subsequently reused.

### Hot filtration (leaching) test procedure

The hot filtration test was conducted to verify the heterogeneous behavior of the catalyst and to probe for the leaching of Ni ions. For this test, a model reaction (Table 2, entry 14) was performed for 3 minutes. Afterward, the reaction mixture was temporarily diluted with a small volume of ethanol (1–2 mL), and then the catalyst was isolated *via* centrifugation. The ethanol was then removed under reduced pressure using a rotary evaporator at 30 °C, thereby restoring near-neat conditions and allowing the reaction to proceed under its original parameters.

## Results and discussion

After preparation, the CS-[BiC-PiC]-NiCl_4_ catalyst was investigated using various techniques, including FT-IR, ^1^H NMR, ^13^C NMR, FESEM, XRD, TGA-DTG, EDS/EDS-map, and ICP. The obtained results are described in the following sections.

### Catalyst characterization

**Fig. 3:**
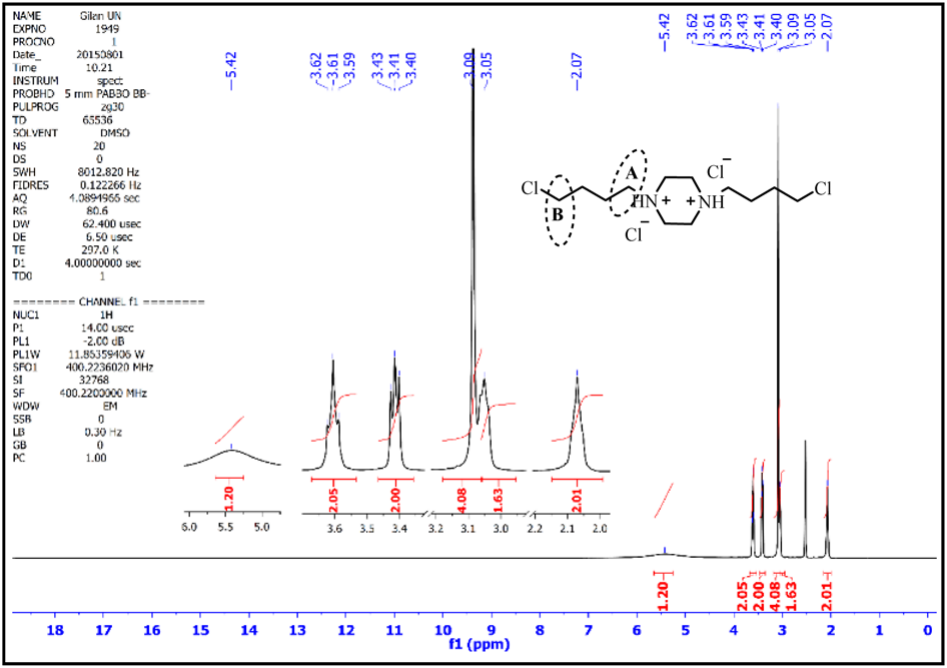
The ^1^HNMR spectra of 1,4-bis(4-chlorobutyl)piperazine-1,4-diium chloride.

The ^1^HNMR spectrum of 1,4-bis(4-chlorobutyl)piperazine-1,4-diium chloride is presented in Fig. 3. As can be seen, two broad peaks with an integral of 2 are observed in the region of 2.07 ppm and 3.05 ppm corresponding to the middle hydrogens of the carbon chain. The hydrogens of the piperazine ring have appeared as single with an integral of 4 in the 3.09 ppm region. The triplet peak with 2 integral and 8 MHz splitting in the 3.41 ppm region corresponds to A hydrogens and the triple peak with 2 integral and 8 MHz splitting in the 3.61 ppm region corresponds to B hydrogens. In this spectrum, the peak corresponding to the NH group is observed as a broad one with an integral of 1 in the area of 5.42 ppm.^49^

**Fig. 4:**
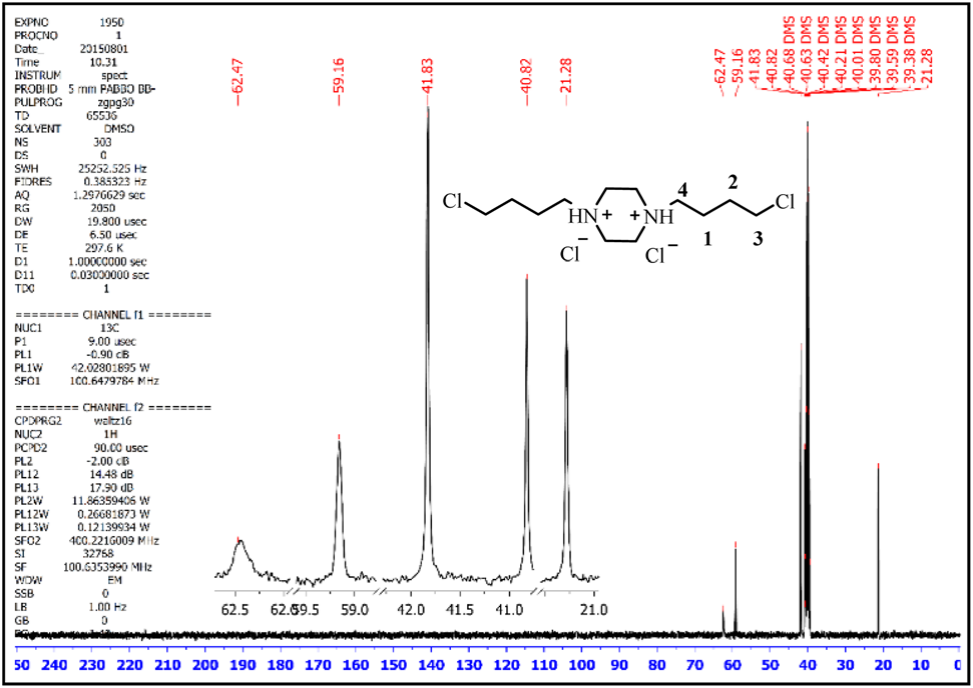
The ^13^CNMR spectra of 1,4-bis(4-chlorobutyl)piperazine-1,4-diium chloride.

In the ^13^CNMR spectrum of the prepared ionic liquid, the corresponding peaks appeared with the correct number and appropriate chemical shift. In this spectrum, the peak of 21.28 ppm corresponds to the carbon number 1, the peak of 40.82 ppm corresponds to the carbon number 2, the peak of 41.83 ppm corresponds to the carbons of the piperazine ring, the peak of 59.16 ppm corresponds to the carbon number 4 and the peak area of 62.47 ppm corresponds to the carbon number 3 (Fig. 4).^49^

**Fig. 5:**
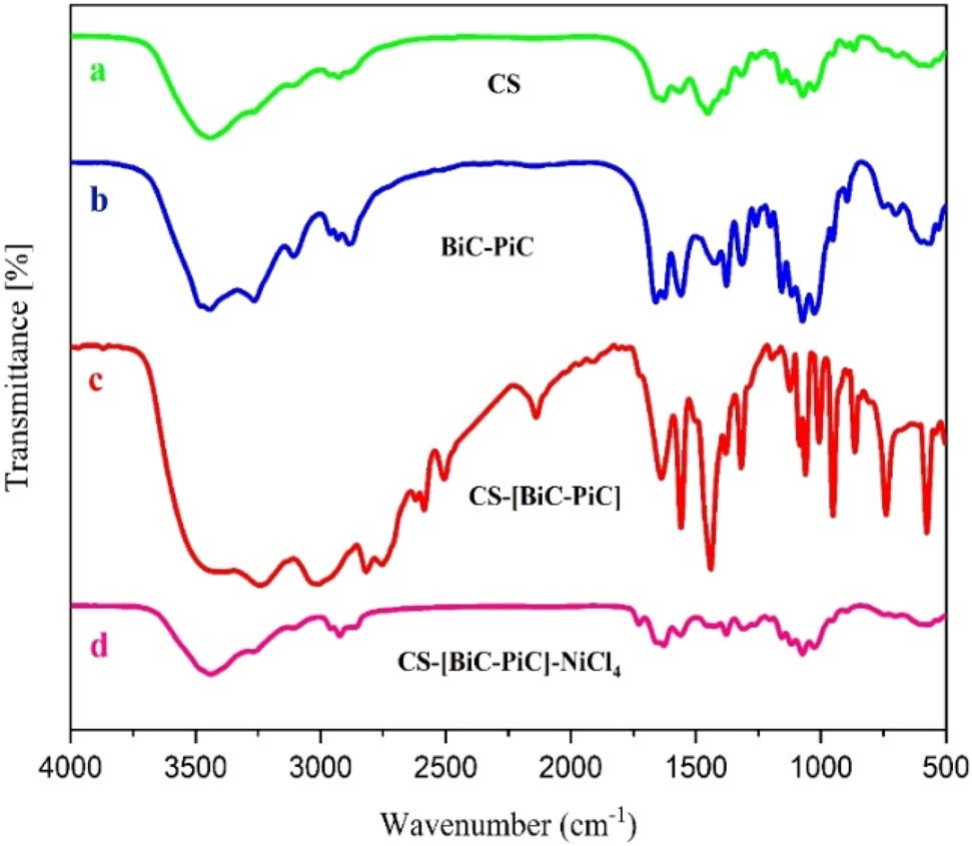
The FT-IR spectra of CS (a), BiC-PiC (b), CS-[BiC-PiC] (c) and CS-[BiC-PiC]-NiCl_4_ (d).

The infrared spectra of CS, BiC-PiC, CS-[BiC-PiC] and CS-[BiC-PiC]-NiCl_4_ are presented and compared in Fig. 5. In the FT-IR spectrum of CS, the broad peak appearing in the range of 3200–3600 cm^−1^ is related to the –OH and –NH functional groups. The peak observed at 2929 cm^−1^ in this spectrum results from the symmetric and asymmetric stretching vibrations of the C–H bond. In addition, the absorption bands observed at 1630 cm^−1^ and 1593 cm^−1^ are assigned to the residual carbonyl acetyl amides and the N–H bending vibrations, respectively. In the CS spectrum, the C–O bond stretching vibrations appear at 1028 cm^−1^, 1073 cm^−1^, and 1157 cm^−1^.^50^

In the BiC-PiC spectrum, the peak observed at 2819 cm^−1^ is assigned to the C–H stretching vibrations in both the piperazine ring and the butyl groups. The broad peak between 2800–3400 cm^−1^ results from the formation of an ammonium salt following the reaction of piperazine with 1,4-dichlorobutane. Additionally, the peak at 577 cm^−1^ corresponds to the stretching vibrations of the C–Cl bond. In the CS-[BiC-PiC] spectrum, the peaks in the region of 2800–3500 cm^−1^ are as a result of the addition of the ionic liquid to the CS substrate. Also, in the FT-IR spectrum of the prepared catalyst, the reaction between NiCl_2_ and CS-[BiC-PiC] leads to a more rigid molecular structure. This increased rigidity restricts molecular vibrations, resulting in reduced peak intensity in the final complex, which can be a result of the addition of nickel chloride to the substrate.

**Fig. 6:**
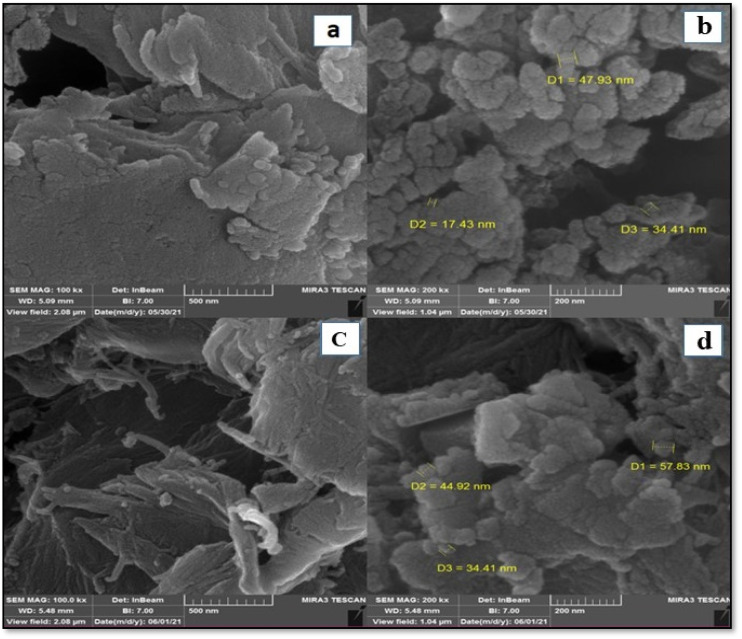
FESEM images of CS (a and b) and CS-[BiC-PiC]-NiCl_4_ (c and d).

The size distribution, particle shape, and surface morphology of CS and CS-[BiC-PiC]-NiCl_4_ were analyzed using field emission scanning electron microscopy (FESEM) (Fig. 6). These images indicate that CS has a smooth and dense surface with minimal roughness and lacks notable three-dimensional features, a result of its polymeric structure. In contrast, the catalyst demonstrates a more three-dimensional surface compared to CS, which can be attributed to changes in the substrate's surface following the reaction of the ionic liquid with the free amino groups on the CS polymer.

**Fig. 7:**
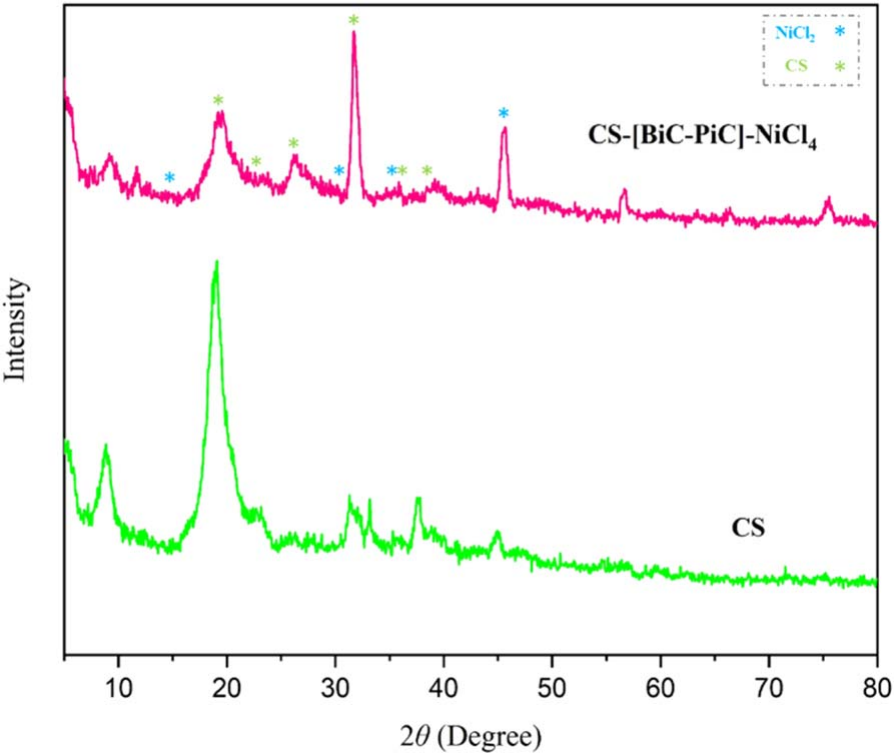
XRD patterns of CS and CS-[BiC-PiC]-NiCl_4_.

The XRD pattern of CS and CS-[BiC-PiC]-NiCl_4_ is shown in Fig. 7. In this study, the XRD pattern of CS shows the characteristic peaks in the 2*θ* region of 10 and 20.^51^

Based on its intra- and intermolecular hydrogen bonds CS has a remarkable crystalline structure compared to most carbohydrate polymers.^52^

After the modification of the surface of the substrate by ionic liquid and involving amino groups, these strong hydrogen bonds are reduced, which resulted in the weakness of the characteristic peaks of CS, indicating the reduction of its crystalline nature. In addition, the presence of these specific peaks in the XRD pattern of the catalyst confirm the stability of the structure and skeleton of CS even after chemical modification.^53^ The appearance of other peaks is due to the presence of organic moieties in the structure of the catalyst. An increase in peak intensity at specific angles indicates enhanced crystallinity or a more organized atomic structure in the resulting complex. This improvement may be attributed to the integration of [BiC-PiC]-NiCl_4_ into the CS matrix. Conversely, the decreased intensity of other peaks could suggest partial amorphization or disruption of the original crystalline structure of CS due to the complex formation.

The XRD pattern of CS-[BiC-PiC]-NiCl_4_ exhibits seven peaks at 2*θ* around 19.42, 23.74, 26.27, 31.71, 35.58, and 38.05, corresponding to the Miller indices (102), (220), (013), (241), (242), and (124) for the CS standard pattern (JCPDS: 00-039-1894). This confirms the presence of CS in the prepared ionic liquid. Moreover, the peaks observed at 2*θ* = 15.1°, 30.3°, 35.6°, and 45.2° are associated with the Miller indices (003), (006), (101), and (104) of NiCl_2_, which corresponds to the JCPDS code 01-073-1646.

Furthermore, the crystallinity degree of CS-[BiC-PiC]-NiCl_4_ was quantified by calculating the ratio of the integrated area under the crystalline peaks to the total diffraction area. The average crystallite size was determined using the Scherer equation, *D* = (*kλ*)/(*β* cos *θ*), where *D* is the crystallite size (nm), *k* is the shape factor (0.9), *λ* is the X-ray wavelength (0.154 nm), *β* is the full width at half maximum (FWHM in radians), and *θ* is the Bragg diffraction angle (radians). The most intense peak in the XRD pattern was used for the calculation. Based on this analysis, the average crystallite size for CS-[BiC-PiC]-NiCl_4_ was found to be 18.46 nm.

**Fig. 8:**
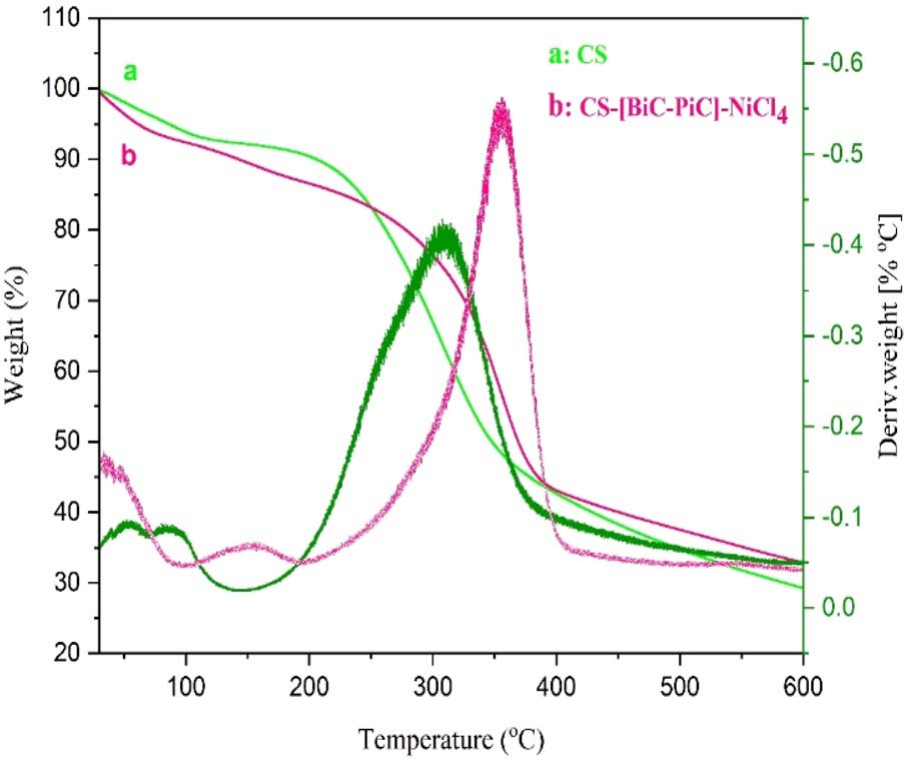
TGA diagrams of CS and CS-[BiC-PiC]-NiCl.

To obtain more information about their thermal stability, the TGA/DTG analysis of CS-[BiC-PiC]-NiCl_4_ and CS, have been compared in Fig. 8. In the curve of CS, the initial weight loss (7%) that occurs up to 150 °C is due to the loss of water absorbed by grafting. But CS-[BiC-PiC]-NiCl_4_ loses at least a few percent less water than CS under the same conditions (up to 150 °C).

The second stage of weight loss for CS starts at 250 °C and only 5% weight is lost up to 300 °C, showing that CS-[BiC-PiC]-NiCl_4_ is more stable in this temperature range. The main degradation step, which is attributed to the probable destruction of the ionic liquid moiety, occurs at around 300 °C and at lower temperatures for the CS-[BiC-PiC]-NiCl_4_ composite.^54^

**Fig. 9:**
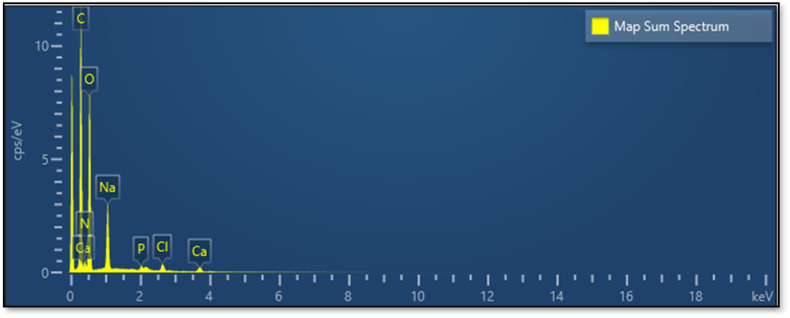
EDS spectrum of CS.

**Fig. 10:**
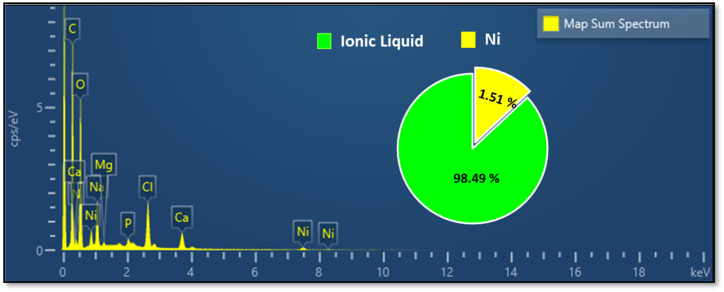
EDS spectrum and ICP of CS-[BiC-PiC]-NiCl_4_.

The elemental composition of the CS substrate and the synthesized catalyst (CS-[BiC-PiC]-NiCl_4_) was determined using Energy Dispersive X-ray Spectroscopy (EDS). The EDS data confirmed the presence of all key incorporation of the intended elements (C, N, O, Ni, and Cl) into the catalyst, while additional elements (Na, Mg, Ca, and P) detected by EDS were attributed to the natural chitosan substrate (Fig. 9 and 10). Furthermore, Inductively Coupled Plasma (ICP) analysis determined the nickel loading in CS-[BiC-PiC]-NiCl_4_ to be 1.51% (Fig. 10).

**Fig. 11:**
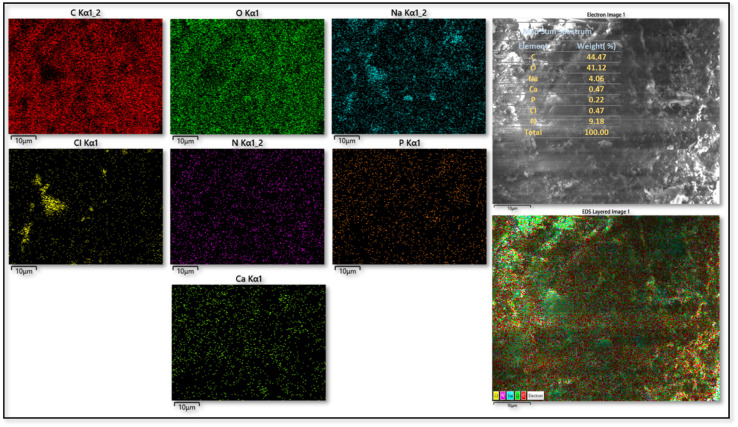
EDS-map analysis of CS.

**Fig. 12:**
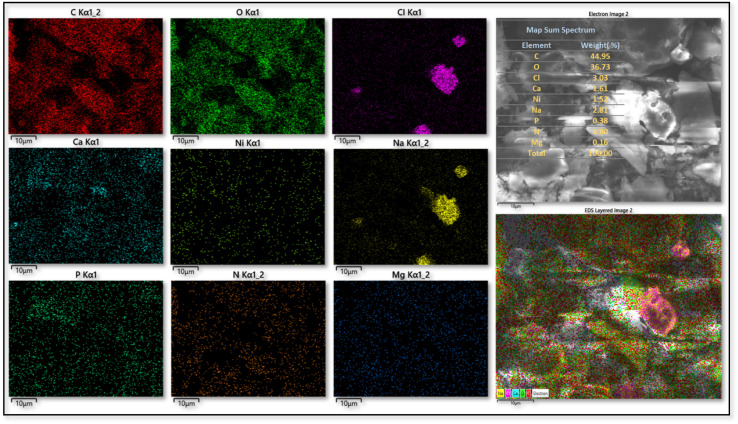
EDS-map analysis of CS-[BiC-PiC]-NiCl_4_.

Beyond identifying elemental composition *via* EDS, the distribution of elements was assessed using elemental mapping analysis (EDS-map). The resulting maps demonstrated a uniform distribution for the elements present in CS (C, N, O, P, Ca, Na, Cl) and the catalyst (C, N, O, Na, Ca, Mg, P, Ni, Cl), which indicates a highly homogeneous structure and strong interactions between the components (Fig. 11 and 12).

### Catalytic activity

After fully characterizing the new catalyst and confirming its structure, it is concluded that it can be an efficient promoter for acid-catalyzed transformations, so its catalytic activity was examined in the synthesis of 2,3-dihydroquinazoline-4(1*H*)-ones *via* two methods as a model of these types of reactions. For this purpose, initially, the reaction of isatoic anhydride, 4-chlorobenzaldehyde, and NH_4_OAc, in 1 : 1 : 2 M ratio, in the presence of the CS-[BiC-PiC]-NiCl_4_ catalyst was studied, and the effect of various parameters, including the amounts of the catalyst, temperature, and solvent, was investigated on it.

**Scheme 3:**
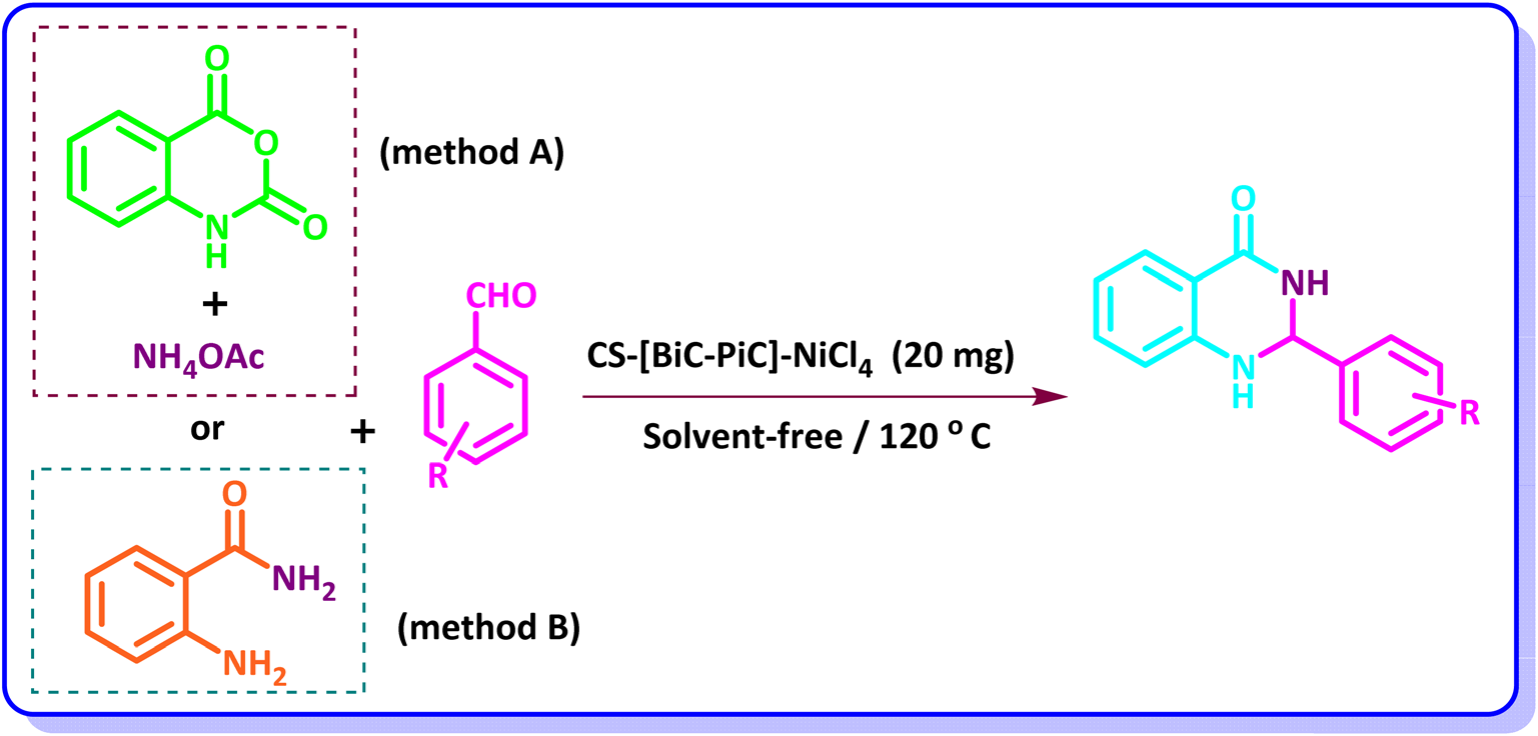
Synthesis of 2,3-dihydroquinazolin-4(1*H*)-ones using CS-[BiC-PiC]-NiCl_4_ as a catalyst.

As could be seen, the best results in terms of time and efficiency are obtained when the reaction is carried out in the presence of 20 mg of the catalyst, at a temperature of 120 °C and in the absence of solvent (Table 1, entry 7) (Scheme 3). The obtained results also demonstrated that the reaction progress was negligible under the remaining conditions.

**Table 1:** Optimization of reaction conditions for the synthesis of 2,3-dihydroquinazolin-4(1*H*)-ones using CS-[BiC-PiC]-NiCl_4_

Entry	Catalyst (mg)	Temperature (°C)	Solvent	Time (min)	Conversion (%)
1	20	Reflux	H_2_O	20	20
2	20	Reflux	EtOH	50	50
3	20	80	H_2_O/EtOH	30	30
4	20	120	DMF	50	25
5	20	100	Solvent-free	30	80
6	20	120	Solvent-free	4	100
7	8	120	Solvent-free	40	70
8	—	120	Solvent-free	60	20

Further studies showed that when 4-chlorobenzaldehyde was reacted with 2-aminobenzamide (method B) the above-mentioned conditions also lead to the best results. So the two methods were performed under the same conditions (Scheme 3).

To establish the scope and generality of the selected reactions, we extended our studies using a diverse range of aromatic aldehydes as variable substrates, and the desired substituted quinazolines were obtained in excellent yields (Table 2). These data show that the electron-withdrawing or electron-donating groups on aromatic rings appear to have a slight effect on the reaction time and efficiency. It is important to say that all reactions proceeded efficiently to the target molecules in high to excellent yields within very short reaction times, and no side products were observed (Table 2).

**Table 2:** Synthesis of 2,3-dihydroquinazolin-4(1*H*)-one derivatives in the presence of CS-[BiC-PiC]-NiCl_4_^a^

Entry	Product	Method		Melting point (°C)	
		Method A	Method B		
		Time (min)	Time (min)		
		Yield^b^ (%)	Yield^b^ (%)	Observed	Reported [ref.]
1	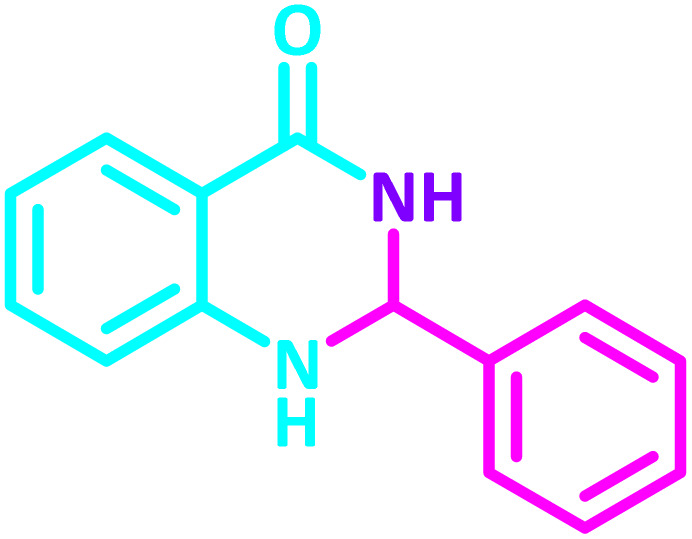	3	4	204–206	215–217 [55]
		95	95		
2	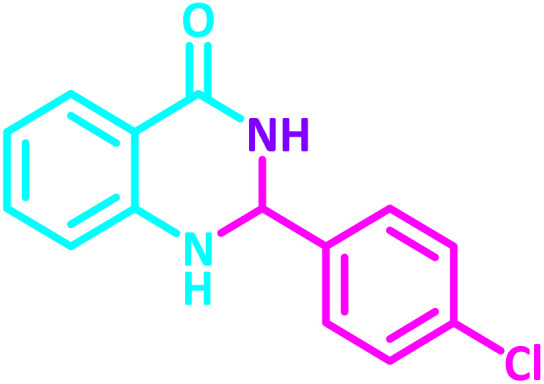	5	5	204–206	201–203 [36]
		94	96		
3	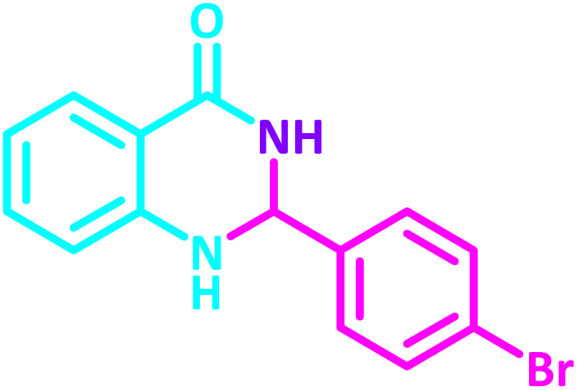	6	5	203–204	203–205 [56]
		96	95		
4	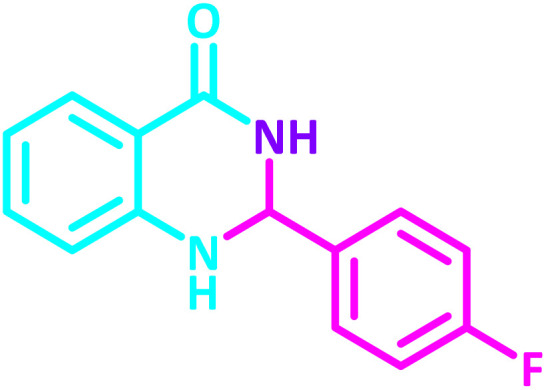	3	3	201–203	199–200 [28]
		94	95		
5	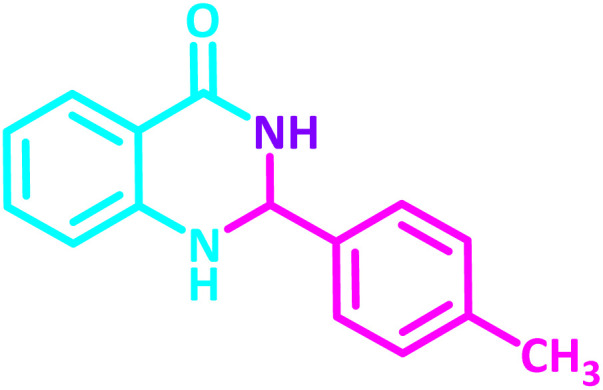	3	3	232–234	230–232 [41]
		96	93		
6	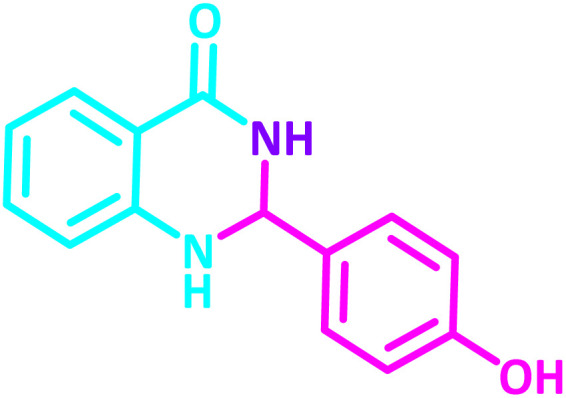	5	4	279–281	278–280 [57]
		94	93		
7	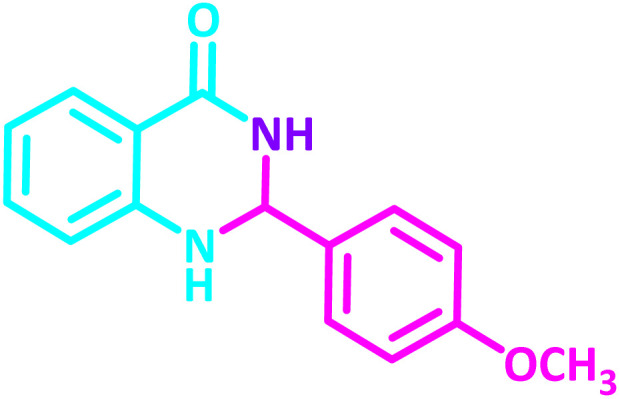	2	5	190–191	188–191 [58]
		95	94		
8	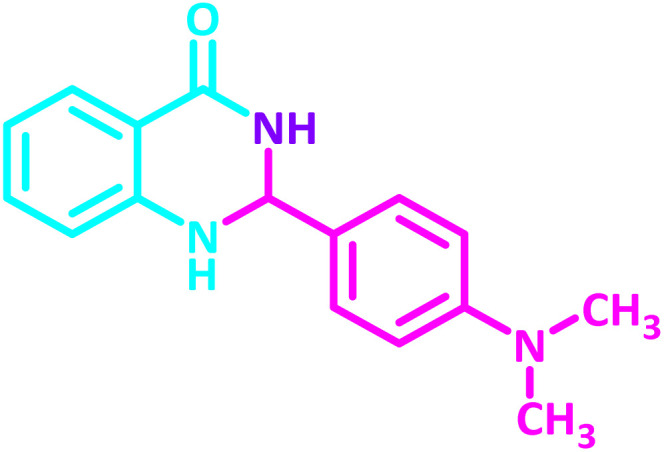	3	4	212–214	214–216 [59]
		97	96		
9	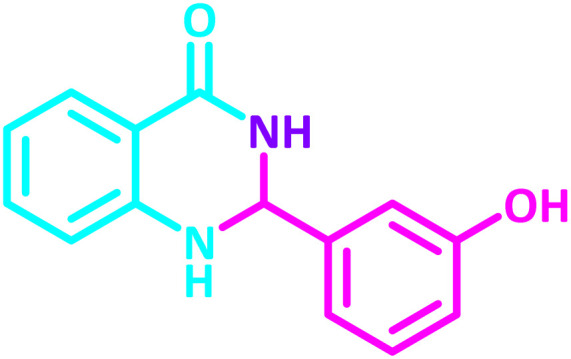	6	5	232–234	224–226 [60]
		97	96		
10	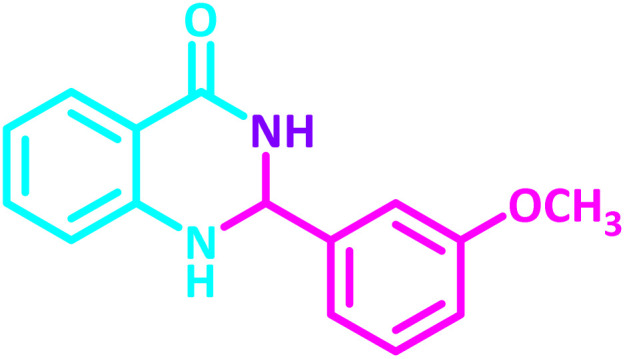	6	6	200–202	202–205 [59]
		94	95		
11	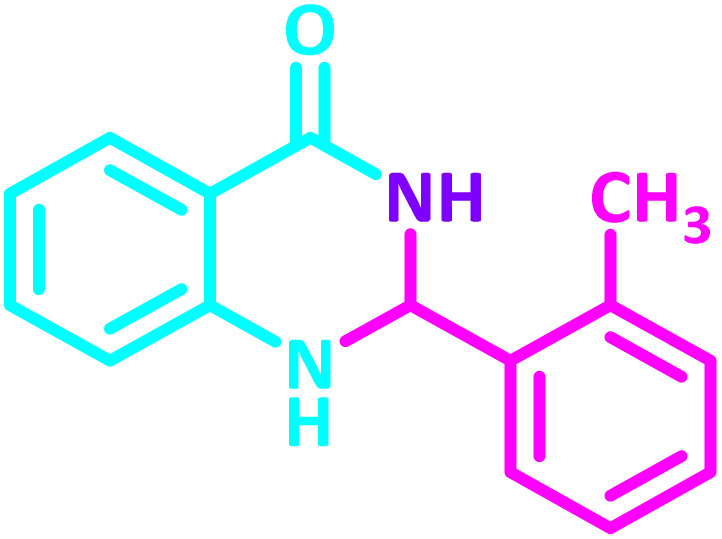	3	7	184–186	187–189 [61]
		95	94		
12	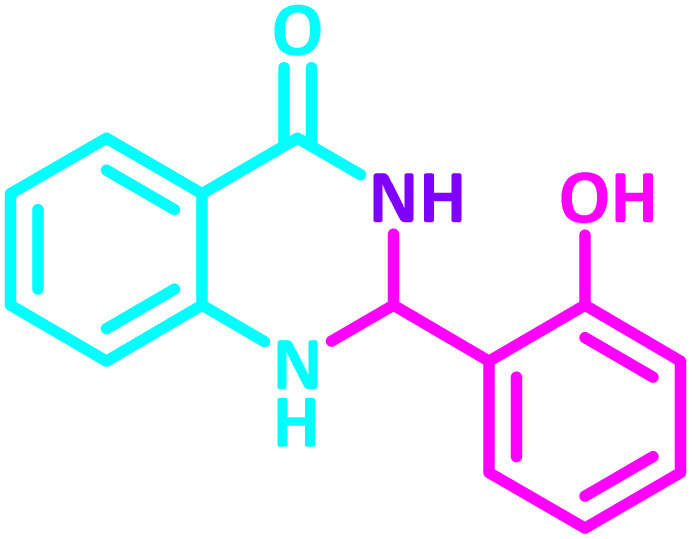	3	4	197–199	208–209 [60]
		95	95		
13	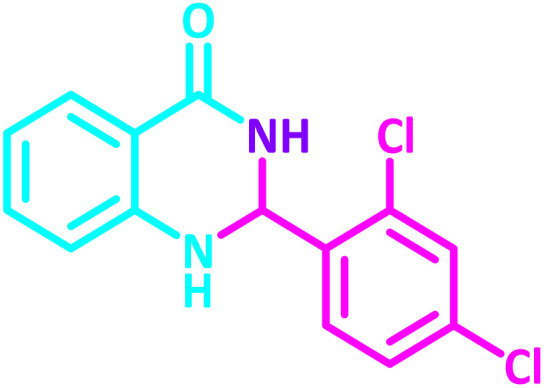	8	4	167–169	208–209 [59]
		94	96		
14	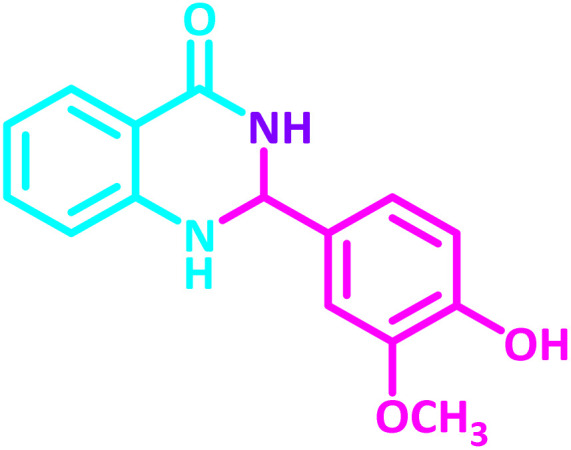	7	2	211–213	213–216 [62]
		93	94		
15	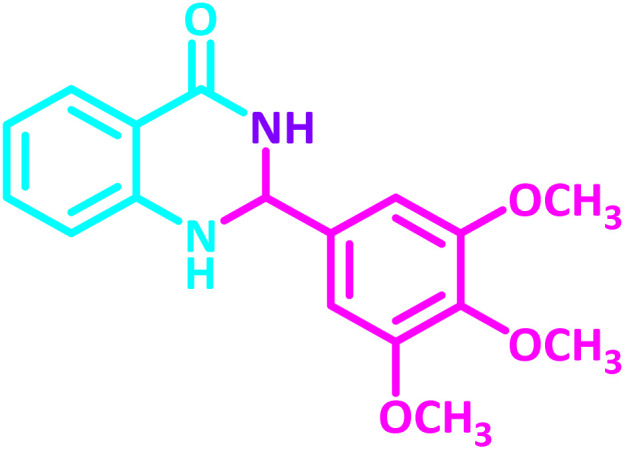	4	4	181–183	185–187 [41]
		94	95		

a Reaction condition: isatoic anhydride (1 mmol), ammonium acetate (2 mmol), aldehydes (1 mmol) or 2-aminobenzamide (1 mmol), aldehydes (1 mmol) and CS-[BiC-PiC]-NiCl_4_ under solvent-free conditions at 120 °C.

b Isolated yields.

**Scheme 4:**
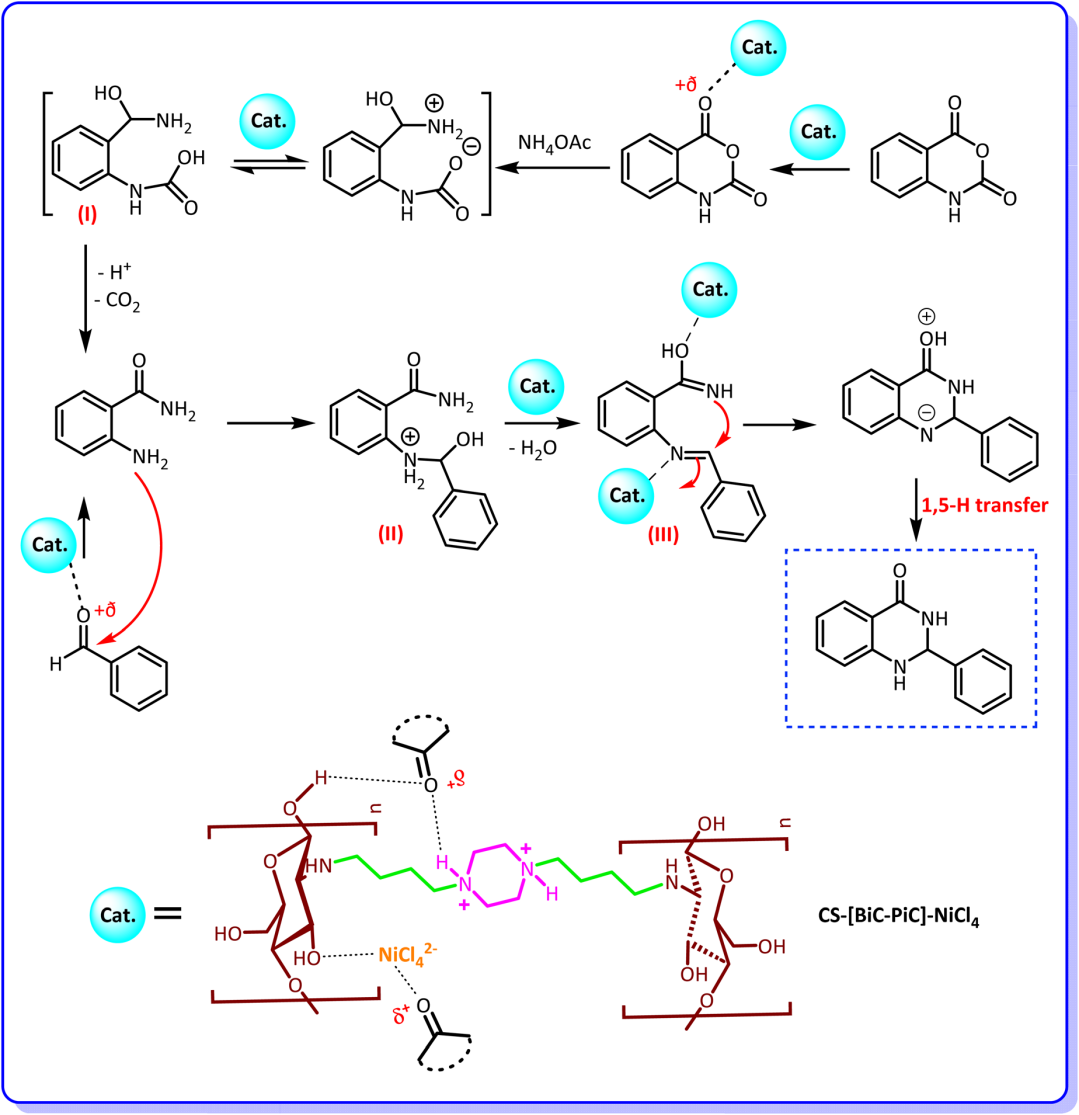
The proposed mechanism for the synthesis of 2,3-dihydroquinazolin-4(1*H*)-ones in the presence of CS-[BiC-PiC]-NiCl_4_.

The proposed mechanism for the synthesis of quinazolin-4(3*H*)-one derivatives in the presence of the introduced catalyst is shown in Scheme 4. Based on this mechanism, the nickel-based catalyst, as a Lewis acidic agent, plays the crucial role of activating the carbonyl group of isatoic anhydride. The Ni^2+^ ion has vacant orbitals that accept electron pairs from the carbonyl oxygen, forming a coordination bond. This bond weakens the carbon–oxygen double bond and makes the carbonyl carbon more electrophilic. As a result, the subsequent amine nucleophilic attack and cyclization step occur much more easily. The second step of this mechanism involves the nucleophilic attack of ammonium acetate on isatoic anhydride, leading to the formation of the activated intermediate (I). Through subsequent reactions, including the release of acidic hydrogen and carbon dioxide, 2-aminobenzamide is obtained. Then, the activated carbonyl group of the aldehyde is attacked by 2-aminobenzamide, through which the intermediate (II) is formed. Removal of a molecule of water from this intermediate can lead to the intermediate (III) from which the desired product is formed by intramolecular nucleophilic attack of the NH group on the imine carbon and finally by 1,5-hydrogen transfer.^53^ It should be mentioned that in the case of method B, the reaction begins by the nucleophilic attack of 2-aminobenzamide on the activated aldehydes.^63,64^

### DFT-based mechanistic justification of CS-[BiC-PiC]-NiCl_4_ catalytic system

To investigate the conceptual nature of the proposed catalytic mechanism and identify which structural motifs in the CS-[BiC-PiC]-NiCl_4_ catalytic system constitute the most active sites, a comprehensive density functional theory (DFT) investigation was conducted to provide molecular-level and electronic justification for the role of chitosan in stabilizing and activating the Ni catalytic center. Rather than relying solely on schematic representation, the present analysis integrates optimized geometries, molecular electrostatic potential (MEP) mapping, quantum theory of atoms in molecules (QTAIM), and reduced density gradient (RDG) analyses to elucidate the nature of metal–support interactions.

The performed DFT calculations were intentionally focused on the catalyst stabilization and activation domain, namely the interaction between the Ni center and chitosan functional groups. The aim was not to compute the full catalytic cycle but to determine which parts of the catalyst structure are electronically and topologically responsible for catalytic competence. The structural motifs considered in the optimized catalyst contain:

A square-planar NiCl_4_ core as the primary catalytic center.

Hydroxyl (–OH) and amino (–NH_2_) groups from chitosan serve as electron-donating anchoring sites.

A secondary network of van der Waals and hydrogen-bonding interactions is provided by the polymeric matrix.

Chitosan, a nitrogen- and oxygen-rich biopolymer, presents multiple coordination sites capable of engaging with transition metal centers through both covalent and noncovalent interactions. Deciphering these interactions is crucial for rationalizing the enhanced performance and stability of Ni catalysts supported on such biomass-derived matrices. Accordingly, the DFT study was designed to examine: (i) the electronic polarization induced upon Ni–chitosan contact, (ii) the topological character of the Ni⋯O/N interactions, and (iii) the interplay of attractive, dispersive, and steric effects within the local catalytic environment.^5,6,65^

The ESP serves as a critical visualization tool, revealing the spatial distribution of electrostatic charge around the system and delineating electron-rich from electron-deficient regions.

Following the standard ESP color scale:

Red denotes areas of negative electrostatic potential, corresponding to high electron density.

Green/yellow signifies regions of near-neutral potential.

Blue represents positive potential, highlighting electron-deficient sites.

**Fig. 13:**
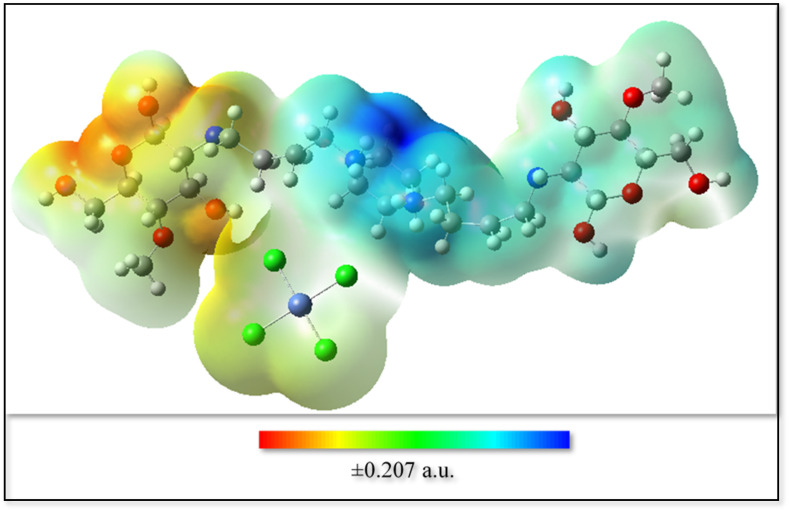
Molecular electrostatic potential CS-[BiC-PiC]-NiCl_4_ a.u.

To gain deeper insight into the electronic features governing the Ni–chitosan interaction, the Molecular Electrostatic Potential (MEP) was mapped onto the electron density surface (Fig. 13). The ESP surface exhibits pronounced negative potential regions (blue) localized around electronegative atoms, particularly oxygen and nitrogen atoms of the chitosan backbone, indicating electron-rich sites capable of coordinating or interacting with metal centers.

In contrast, regions of positive electrostatic potential (yellow to red) are mainly distributed around hydrogen atoms and the vicinity of the Ni center, reflecting electron-deficient domains that can act as electrophilic sites. This complementary charge distribution strongly favors electrostatic attraction and coordination-assisted interactions between the Ni center and the electron-rich functional groups of chitosan, such as hydroxyl and amino moieties.

The observed polarization indicates a significant charge redistribution upon complexation, which contributes to the stabilization of the active metal site. Notably, the spatial overlap between electron-rich regions of the support and electron-deficient regions near the Ni center provides a clear electronic rationale for the anchoring effect suggested by the RDG and QTAIM analyses.

Collectively, the ESP analysis, complemented by noncovalent interaction and topological studies, supports a mechanism of chitosan-assisted electronic stabilization of the Ni active site. This stabilization, augmented by secondary interactions from the ionic liquid, is fundamental to the enhanced catalytic performance observed experimentally.

### DFT-based electronic justification of the catalytic cycle

To move beyond a purely conceptual proposal, we analyzed the wavefunction of the Ni-containing catalytic system. The optimized Ni–Cl core exhibits a square-planar NiCl_4_ arrangement with nearly identical Ni–Cl distances (∼2.14 Å) and characteristic 90°/180° angles, consistent with a coordinatively defined Ni center. Frontier orbital inspection shows that the highest-lying occupied orbitals have dominant Ni d-character (≈99% Ni contribution for the top occupied level; ∼−3.68 eV) along with nearby Cl p-dominated and mixed Ni/Cl levels. This electronic structure supports a Ni-centered redox/coordination role in the proposed cycle and rationalizes how ligand/medium effects can modulate electron transfer and activation steps.

### AIM/QTAIM analysis of Ni–O coordination

To rationalize the anchoring interaction between the Ni center and the chitosan matrix, QTAIM analysis was performed on the Ni⋯O(OH) contact (Table 3). A bond critical point (BCP) was identified between Ni and the alcoholic oxygen, with *ρ*(BCP) = 0.01669 a.u. and ∇^2^*ρ*(BCP) = −0.01735 a.u. The energy-density descriptors (*G* = 0.01609 a.u., *V* = −0.01484 a.u.) yield *H* = *G* + *V* = +0.00125 a.u. and |*V*|/*G* = 0.92, indicating a predominantly closed-shell/coordination interaction with a limited but non-negligible covalent contribution. This Ni–O coordination supports the proposed mechanistic role of chitosan in stabilizing the active Ni species and facilitating substrate activation.

**Table 3:** QTAIM (AIM) descriptors at the bond critical point (BCP) for the Ni⋯O(OH) interaction in the chitosan matrix^a^

Interaction	BCP coordinates (*x*, *y*, *z*)	*ρ*(BCP)	∇^2^*ρ*(BCP)	*G*(BCP)	*V*(BCP)	*H*(BCP)	|*V*|/*G*	Qualitative assignment
Ni⋯O(OH) (chitosan)	(−9.8078, −1.8314, −0.5220)	0.0166	−0.0173	0.0160	−0.014	0.0012	0.922	Predominantly closed-shell/coordination with limited covalent contribution

a Suggested in-text statement: a BCP is observed for Ni⋯O(OH) with *ρ* = 0.01669 a.u. and ∇^2^*ρ* = −0.01735 a.u.; *H* > 0 and |*V*|/*G* ≈ 0.92 indicate a predominantly coordination (closed-shell) interaction with partial covalent character. Units are atomic units (a.u.). *H* is the total energy density (*H* = *G* + *V*). The ratio |*V*|/*G* is provided as a qualitative indicator of interaction character.

**Fig. 14:**
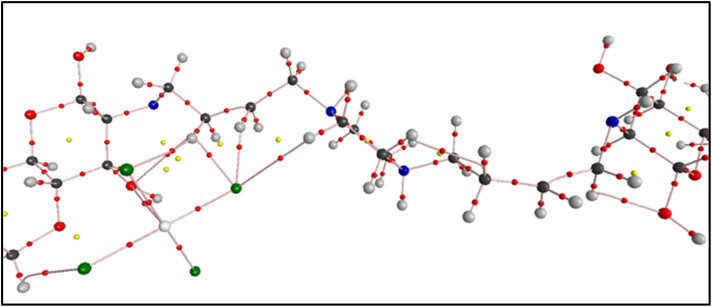
QTAIM (AIM) molecular graph of the Ni–chitosan system, showing bond paths and critical points of the electron density. The bond path and the corresponding bond critical point (BCP) between Ni and the alcoholic oxygen of chitosan indicate a real Ni⋯O coordination/anchoring interaction. Small red points denote BCPs, and yellow points denote ring critical points (RCPs) (where present).

Fig. 14 shows the QTAIM molecular graph of the Ni–chitosan system. The presence of a bond path and an associated bond critical point (BCP) between Ni and the alcoholic oxygen of chitosan supports a genuine coordination/anchoring interaction. Together with the BCP descriptors reported in Table 3 (*ρ*(BCP) = 0.01669 a.u., ∇^2^*ρ*(BCP) = −0.01735 a.u., *H* > 0 and |*V*|/*G* ≈ 0.92), the interaction can be classified as predominantly closed-shell/coordination with a limited but non-negligible covalent contribution. This anchoring effect rationalizes the stabilization of the Ni active site by the chitosan matrix and strengthens the mechanistic proposal beyond a purely conceptual scheme.

To further characterize the noncovalent interactions between the Ni center and the chitosan fragment, reduced density gradient (RDG) analysis was carried out. The RDG scatter plot (RDG *versus* sign(*λ*_2_)*ρ*) exhibits a pronounced spike around sign(*λ*_2_)*ρ* ≈ 0, which is a typical fingerprint of weak dispersive van der Waals interactions. These interactions play an important role in stabilizing adsorbed intermediates and maintaining the structural integrity of the Ni–chitosan framework.

Moreover, the presence of features extending into the negative sign(*λ*_2_)*ρ* region indicates attractive interactions within the system, which can be attributed to coordination-assisted contacts and hydrogen-bond-like interactions, such as Ni⋯O(OH) interactions between the Ni center and hydroxyl groups of chitosan. These attractive interactions contribute to anchoring the Ni active site onto the chitosan matrix and facilitate electronic stabilization of the catalytic center.

In contrast, regions located at positive sign(*λ*_2_)*ρ* values correspond to steric repulsion, reflecting spatial constraints imposed by the chitosan backbone that help regulate the orientation of interacting species.

**Fig. 15:**
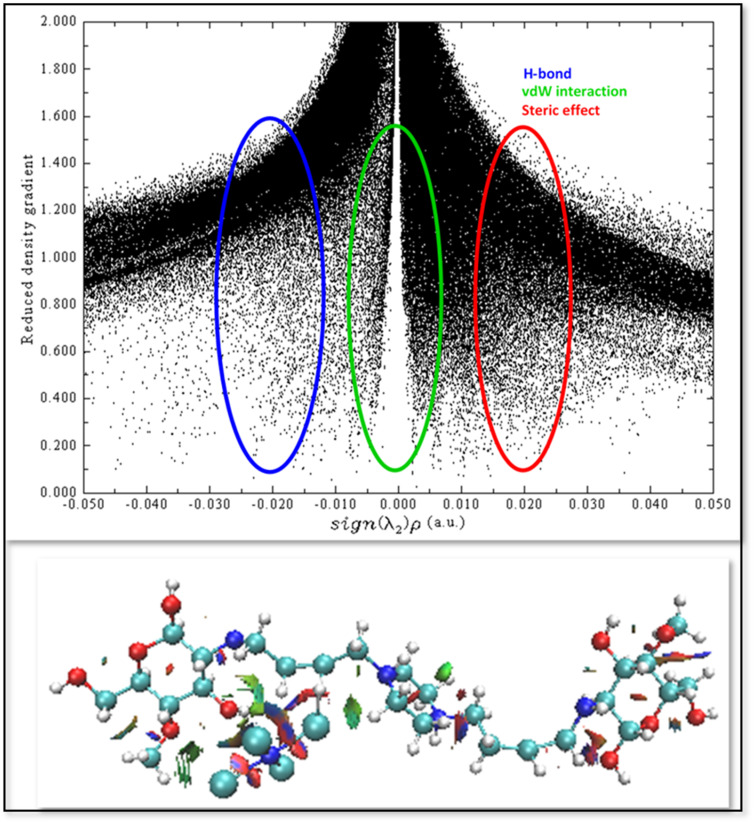
Reduced density gradient (RDG) *versus* sign(*λ*_2_)*ρ* plot and corresponding noncovalent interaction (NCI) isosurfaces for the Ni–chitosan catalytic system. Blue, green, and red regions represent hydrogen bonding interactions, van der Waals forces, and steric repulsion, respectively. The analysis highlights the synergistic interactions between Ni active sites and chitosan functional groups, which contribute to catalyst stabilization and enhanced reaction kinetics.

The coexistence of dispersive, attractive, and steric interactions revealed by RDG analysis is fully consistent with the QTAIM results, including the identification of bond critical points (BCPs) and associated topological parameters. Together, these complementary analyses provide strong evidence for a chitosan-assisted stabilization mechanism of the Ni active site, thereby supporting the proposed interaction model and its relevance to the observed catalytic performance (Fig. 15).

Based on the preceding analysis of electronic and structural descriptors, the catalytic system exhibits a clearly defined hierarchy of active sites. The nickel center functions as the primary active site, serving as the indispensable redox and coordination hub. Directly supporting this core is the secondary active site: the hydroxyl oxygen of the chitosan framework, which coordinates the nickel ion. Further modulating reactivity are tertiary sites, comprising adjacent chitosan-based hydrogen bond donors and the ligand's inherent steric framework, which collectively shape the local environment and fine-tune catalytic activity.

The efficiency of the presented method can be shown by the comparison of the obtained results from the synthesis of 3,2-dihydroquinazoline 4(3*H*)-ones catalyzed by CS-[BiC-PiC]-NiCl_4_ with some other reported catalysts in the literature (Table 4). According to the results, it can be seen that CS-[BiC-PiC]-NiCl_4_ is a superior catalyst than other catalysts in terms of the catalyst amount, reaction conditions, time, and the yield of the products. Table 4 also shows that CS and CS-[BiC-PiC] are also able to accelerate these reactions, but the reaction times are longer than the case of the nickel-based catalyst. This result shows the effect of the change made in the structure of the final catalyst to reach to more catalytic activity.

**Table 4:** Comparison study of the efficiency of the CS-[BiC-PiC]-NiCl_4_ with some of those reported in the literature for the synthesis of 2,3-dihydroquinazolin-4(1*H*)-ones

Entry	Catalyst (mg)	Condition	Time (min)	Yield^a^ (%)	Ref.	Product
1	SBNPSA (100 mg)	EtOH/reflux	180	87	66	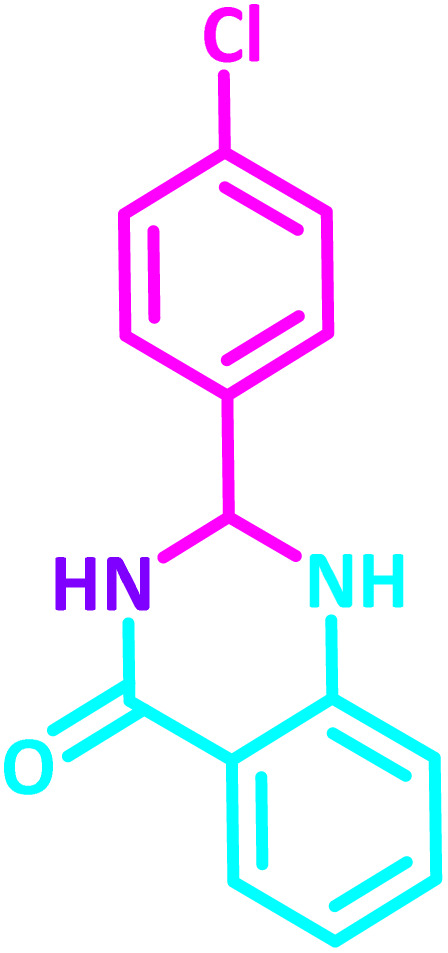
2	VB1 (3 mol%)	EtOH/reflux	180	85	67	
3	SBSSA (5 mg)	EtOH/80 °C	130	90	68	
4	CS (20 mg)	Solvent-free/120 °C	60	87	This work	
5	CS-[BiC-PiC] (20 mg)	Solvent-free/120 °C	20	90	This work	
6	CS-[BiC-PiC]-NiCl_4_ (20 mg)	Solvent-free/120 °C	5	96	This work	

a Isolated yields. SBNPSA: silica-bonded *N*-propylsulfamic acid; VB1: thiamine hydrochloride; SBSSA: silica bonded *S*-sulfonic acid.

**Fig. 16:**
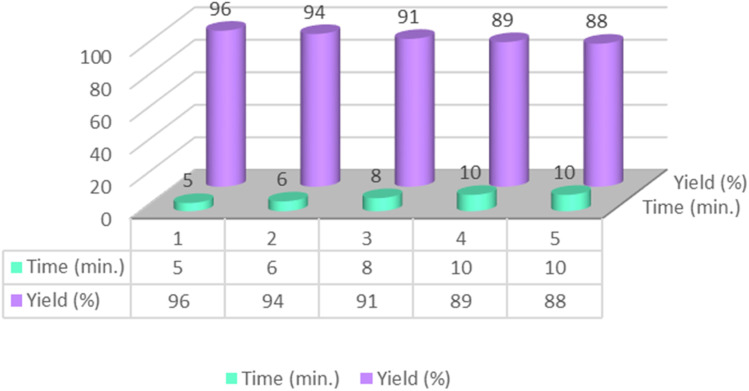
Reusability of the CS-[BiC-PiC]-NiCl_4_ catalyst in the reaction of isatoic anhydride and 4-chlorobenzaldehyde with NH_4_OAC.

**Fig. 17:**
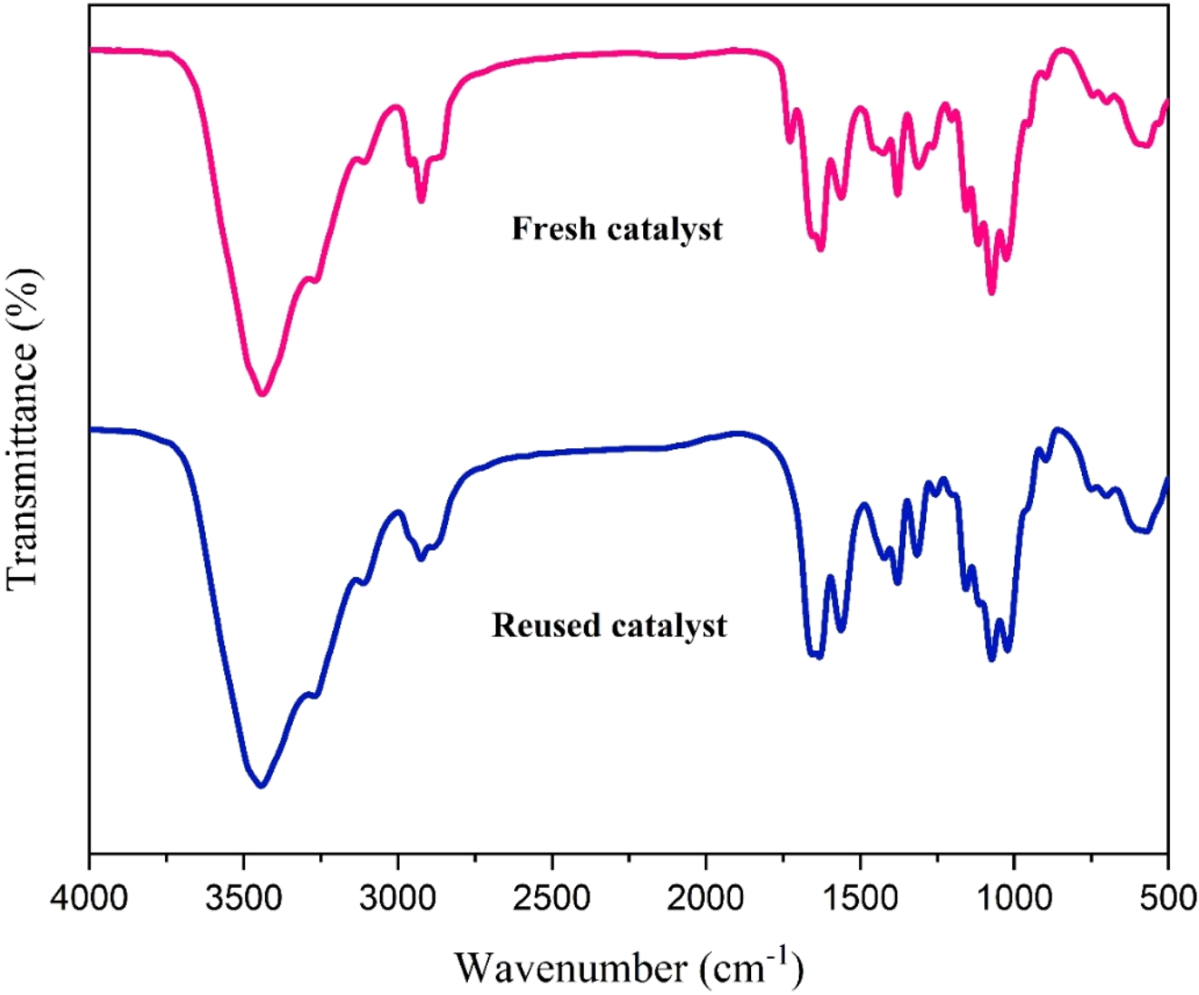
Comparison of the FT-IR spectra of fresh and recycled catalyst after five runs.

**Fig. 18:**
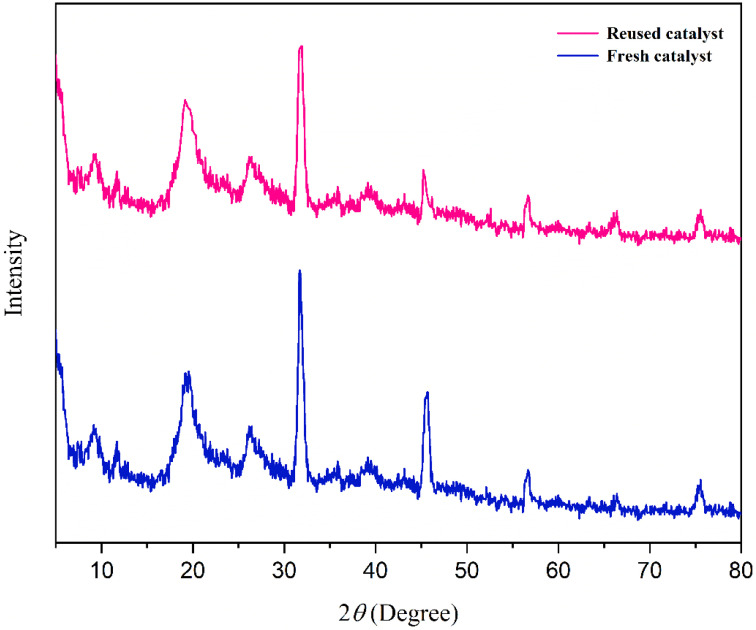
Comparison of the XRD patterns of fresh and recycled catalyst.

The catalyst's reusability was assessed over five consecutive cycles under optimized conditions. As illustrated in Fig. 16, it exhibited excellent stability, retaining high catalytic activity with only a minimal performance loss. This minor loss in activity may be attributed to partial blockage of active sites or minimal catalyst loss during the isolation and washing procedures. Furthermore, comparative FT-IR and XRD studies of the newly prepared catalyst with the recycled catalyst showed no significant degradation under the reaction conditions (Fig. 17 and 18). This was further supported by ICP-OES analysis, which measured a retained nickel content of 1.40% in the recovered catalyst, indicating negligible metal leaching.

To verify the heterogeneous behavior of the catalyst and to probe for the leaching of Ni ions, a hot filtration test was performed under the optimized reaction conditions. After approximately half of the reaction time had elapsed, the catalyst was removed from the reaction mixture by hot filtrate was then allowed to continue under the same reaction conditions in the absence of the solid catalyst. Following the separation, the reaction exhibited no significant conversion for 50 minutes, starkly contrasting with the 7 minute completion time observed with the catalyst. These findings indicate that the catalyst operates heterogeneously, with no considerable leaching of active species into the reaction mixture.

## Conclusions

In summary, this work demonstrates the successful synthesis of a novel heterogeneous catalyst, CS-[BiC-PiC]-NiCl_4_, through the immobilization of a nickel-containing dicationic ionic liquid onto chitosan. This catalyst proved highly effective for the synthesis of 2,3-dihydroquinazolin-4(1*H*)-ones. The structural integrity of the synthesized compound was rigorously confirmed by a comprehensive characterization suite, including ^1^H NMR, ^13^C NMR, FT-IR, XRD, TGA/DTG, FESEM, EDS/EDS-mapping, and ICP-OES, complemented by computational methods. The presented methodology offers significant advantages, including excellent catalyst reusability, short reaction times, solvent-free conditions, a simple workup procedure, high product yields without the need for purification, and facile catalyst separation *via* filtration. The catalyst exhibited remarkable activity and broad substrate scope. Crucially, a hot filtration test confirmed its robust heterogeneous nature, with analysis showing negligible nickel leaching. This stability enabled the catalyst to be recycled for five runs with minimal loss of activity. Consequently, this protocol represents a sustainable, cost-effective, and environmentally benign strategy for the synthesis of pharmaceutically relevant quinazolinone scaffolds. Density functional theory (DFT) calculations were employed to justify the catalytic mechanism and identify the most active structural motifs in the CS-[BiC-PiC]-NiCl_4_ system. Molecular electrostatic potential (MEP) analysis reveals a pronounced negative potential localized on the oxygen and nitrogen atoms of the chitosan (CS) backbone, identifying these regions as preferential anchoring sites. This assignment is substantiated by Quantum Theory of Atoms in Molecules (QTAIM) analysis, which identifies a bond critical point between the nickel center and a chitosan hydroxyl oxygen (*ρ* = 0.01669 a.u.; |*V*|/*G* ≈ 0.92). The associated topological descriptors characterize this interaction as coordination-dominated with partial covalent character. Further analysis *via* the Reduced Density Gradient (RDG) method highlights a network of stabilizing non-covalent interactions, including van der Waals contacts and hydrogen bonds, enveloping the Ni site to form a confined catalytic microenvironment. Collectively, these computational findings demonstrate that chitosan acts not as a passive support but as an integral, electronically active component. Its hydroxyl and amino groups function as key sites that stabilize the Ni center, modulate its charge density, and facilitate substrate activation.

## Conflicts of interest

There are no conflict to declare.

## Supplementary Material

RA-016-D5RA07849K-s001

## Data Availability

No primary research results, software or code have been included and no new data were generated or analysed as part of this article. Supplementary information (SI): FT-IR, ^1^H NMR & ^13^C NMR of new products. See DOI: https://doi.org/10.1039/d5ra07849k.
