# Supplementary material for: CS-[BiC-PiC]-NiCl4: a green and effective heterogeneous catalyst for one-pot synthesis of 2,3-dihydroquinazolin-4(1H)-one derivatives
Source: RSC Adv. 2026 Feb 11;16(9):8337–55. doi: 10.1039/d5ra07849k (PMC12893883; doi:10.1039/d5ra07849k)
Supplement: RA-016-D5RA07849K-s001 [file RA-016-D5RA07849K-s001.pdf]

Supplementary Information (SI) for RSC Advances.
This journal is © The Royal Society of Chemistry 2026

Supporting Information

CS-[BiC-PiC]-$NiCl_4$: As a green and effective heterogeneous catalyst for one-pot synthesis of 2, 3-dihydroquinazolin-4 (1*H*)-one derivatives

Narges Seyedi, Maryam Mousapour Mohammad Rizehbandi and Farhad Shirini [a]*

[a] *Department of Organic Chemistry, College of Chemistry, University of Guilan, Rasht, 41335-19141, Iran, Tel/Fax: +981313233262;*

E-mail: shirini@guilan.ac.ir (and also fshirini@gmail.com)

List of Contents……………………………………………………………………...…Page no

1. General information and procedure ……………………………………………….…...2

2. The spectral data of the selected compounds ………………………………………………3

3. ^{1}H and ^{13}C NMR spectra of compounds…………………………………………….….. 7

1. Experimental section

Materials and measurements

All solvents and chemicals in this study were of analytical grade and purchased from Merck (Munich) and Sigma-Aldrich (Mumbai) Chemical Companies and were used without further purification. Determination of substrate purity and reaction monitoring were accomplished by TLC on silica-gel polygram SILG/UV 254 plates. Yields refer to the isolated products. The products were characterized based on their physical constants, comparison with authentic samples, and FT-IR and NMR spectroscopy. The Fourier-transform infrared spectra (FT-IR) were recorded with a VERTEX 70 (Brucker, Germany) instrument using KBr pellets for solid samples in the range of 4000–400 cm^{-1}. The ^{1}H and ^{13}C NMR were run on a 400 MHz Bruker Avance in DMSO-d6 using TMS as an internal standard. X-ray diffraction (XRD) was performed on an X'Pert Pro instrument (Panalytical Company Netherlands). Field emission scanning electron microscopy (FESEM) was done using a TE-SCAN model Sigma VP (ZEISS Company in Germany). Thermogravimetric analysis (TGA) was performed on a TGA-DTA METTLER TGA/STTA 851 (Swiss). Metal content in the nanocatalyst was analyzed by a thermo scientific inductively coupled plasma (ICP-OES) (ESPECTRO ARCOS).

General method for the synthesis of 2,3-dihydroquinazolin-4(1*H*)-ones (method A)

For the reaction, to a mixture of isatoic anhydride (1 mmol), aryl aldehyde (1 mmol) and ammonium acetate (2 mmol), CS-[BiC-PiC]-$NiCl_4$ (20 mg) was added. Then the reaction mixture was stirred at 120 °C for the appropriate time. Upon completion as indicated by TLC (*n*-hexane:ethyl acetate, 5:2), ethanol (10 mL) was added to the mixture and the catalyst was separated by filtration. After evaporation of the solvent from the filtrate, the corresponding product was obtained with high purity in good to excellent yields.

General method for the 2,3-dihydroquinazolin-4(1*H*)-ones (method B)

A mixture of 2-aminobenzamide (1 mmol) and aryl aldehyde (1 mmol), and CS-[BiC-PiC]-$NiCl_4$ (20 mg) was stirred at 120 °C for the appropriate time. The progress of the reaction was monitored by TLC [*n*-hexane: ethyl acetate (5:2)]. After the completion of the reaction, ethanol (10 mL) was added to the mixture and the catalyst was separated by filtration. After evaporation of the solvent, the desired product was obtained with high purity in good to excellent yields.

The spectral data of the selected compounds are as follow:

2-phenyl-2, 3-dihydroquinazolin-4(1*H*)-one (Table 2, entry 1, ; Fig. S1 and Fig. S2): mp.: 204-206 °C; ^{1}H NMR (500 MHz, DMSO): δ = 8.31 (s, 1H), 7.64 (dd, J = 7.7, 1.1 Hz, 1H), 7.52 (d, J = 7.1 Hz, 2H), 7.45-7.33 (m, 3H), 7.30-7.22 (m, 1H), 7.13 (s, 1H), 6.77 (d, J = 8.0 Hz, 1H), 6.73-6.66 (m, 2H), 5.78 (s, 1H) ppm; ^{13}C NMR (125 MHz, DMSO): δ = 164.08, 148.35, 142.11, 133.79, 128.94, 128.81, 127.83, 127.35, 117.59, 115.43, 114.88, 67.04 ppm.

2-(4-Chlorophenyl)-2,3-Dihydroquinazolin-4(1*H*)-One (Table 2, entry 2, ; Fig. S3 and Fig. S4): m.p.: 204-206 °C, ^{1}H NMR (500 MHz, DMSO): δ = 8.31 (s, 1H), 7.60-7.43 (m, 2H), 7.25-7.22 (m, 4H), 7.12 (s, 1H), 6.68 (dd, J = 15.5, 7.7 Hz, 2H), 5.75 (s, 1H) ppm; ^{13}C NMR (125 MHz, DMSO): δ = 163.88, 147.96, 140.99, 133.80, 133.31, 129.07, 128.66, 127.71, 117.67, 115.22, 114.80, 66.04 ppm.

2-(4-Bromophenyl)-2,3-dihydroquinazolin-4(1*H*)-one (Table 2, entry 3, ; Fig. S5 and Fig. S6): m.p.: 203–204 °C, ^{1}H NMR (500 MHz, DMSO-d6): δ = 8.36 (s, 1 H), 7.64–7.47 (m, 3 H), 7.45 (d, J = 8.4 Hz, 2 H), 7.28–7.17 (m, 1 H), 7.17 (s, 1 H), 6.77 (d, J = 8.0 Hz, 1 H), 6.71–6.68 (m, 1 H), 5.78 (s, 1 H) ppm. ^{13}C NMR (125 MHz, DMSO-d6): δ = 164.08, 148.20, 141.66, 133.99, 131.82, 129.66, 127.95, 122.16, 117.88, 115.51, 114.04, 66.38 ppm.

2-(4-Fluorophenyl)-2,3-Dihydroquinazolin-4(1*H*)-One (Table 2, entry 4, ; Fig. S7 and Fig. S8): m.p.: 201-203 °C; ^{1}H NMR (500 MHz, DMSO): δ = 8.31 (s, 1H), 7.63 (dd, J = 7.7, 1.3 Hz, 1H), 7.57-7.55 (m, 2H), 7.26-7.25 (m, 3H), 7.12 (s, 1H), 6.77 (d, J = 8.0 Hz, 1H), 6.72- 6.69 (m, 1H), 5.80 (s, 1H) ppm; ^{13}C NMR (125 MHz, DMSO): δ = 164.15, 161.72, 148.38, 138.37, 133.93, 129.65, 129.58, 127.94, 117.83, 115.75, 115.58, 115.53, 115.02, 66.52 ppm.

2-(4-Methylphenyl)-2,3-Dihydroquinazolin-4(1*H*)-One (Table 2, entry 5, ; Fig. S9 and Fig. S10): m.p.: 232-234 °C; ^{1}H NMR (500 MHz, DMSO-d6): δ = 8.20 (s, 1H), 7.58 (d, J = 7.3 Hz, 1H), 7.34 (d, J = 7.4 Hz, 2H), 7.27-7.11 (m, 3H), 7.02 (s, 1H), 6.71 (d, J = 7.9 Hz, 1H), 6.64 (t, J = 7.1 Hz, 1H), 5.68 (s, 1H), 2.27 (s, 3H) ppm; ^{13}C NMR (125 MHz, DMSO-d6): δ = 164.14, 148.38, 139.15, 138.20, 133.76, 129.30, 127.82, 127.25, 117.56, 115.46, 114.89, 66.82, 21.21 ppm.

2-(4-Hydroxylphenyl)-2,3-Dihydroquinazolin-4(1*H*)-One (Table 2, entry 6, ; Fig. S11 and Fig. S12): m.p.: 279–281 C; ^{1}H NMR (600 MHz, DMSO-d6): δ = 9.49 (s, 1H, OH), 8.08 (s, 1H, N–H), 7.61 (d, 1H, J=6.96 Hz, Ar–H), 7.30 (d, 2H, J=8.46 Hz, Ar–H), 7.23 (t, 1H, J=6.93 Hz, Ar–H), 6.93 (s, 1H, N–H), 6.77–6.72 (m, 3H, Ar–H), 6.67 (t, 1H, J=7.38 Hz, Ar–H), 5.65 (s, 1H, CH) ppm; ^{13}C NMR (150 MHz, DMSO-d6): δ = 163.7, 157.6, 148.1, 133.1, 131.6, 129.6, 128.2, 127.3, 117.0, 115.3, 114.9, 114.3, 66.6 ppm.

2-(4-Methoxyphenyl)-2,3-Dihydroquinazolin-4(1*H*)-One (Table 2, entry 7, ; Fig. S13 and Fig. S14): m.p.: 190-191 °C; ^{1}H NMR (600 MHz, DMSO-d6): δ = 8.18 (s, 1H), 7.60 (d, J = 7.5 Hz, 1H), 7.41 (d, J = 8.5 Hz, 2H), 7.24 (t, J = 7.6 Hz, 1H), 7.01 (s, 1H), 6.94 (d, J = 8.7 Hz, 2H), 6.73 (d, J = 8.1 Hz, 1H), 6.67 (t, J = 7.4 Hz, 1H), 5.70 (s, 1H), 3.75 (s, 3H) ppm; ^{13}C NMR (125 MHz, DMSO-d6): δ = 164.35, 159.94, 148.46, 133.99, 133.76, 128.72, 127.86, 117.60, 115.37, 114.92, 114.15, 66.79, 55.70 ppm.

2-(4-(Dimethylamino)phenyl)-2,3-Dihydroquinazolin-4(1*H*)-One (Table 2, entry 8, ; Fig. S15 and Fig. S16): m.p.: 212-214 °C; ^{1}H NMR (500 MHz, DMSO-d6): δ = 8.11 (s, 1H), 7.62 (d, J = 7.7 Hz, 1H), 7.32 (d, J = 8.3 Hz, 2H), 7.24 (t, J = 7.6 Hz, 1H), 6.95 (s, 1H), 6.71 (dt, J = 14.8, 5.7 Hz, 4H), 5.65 (s, 1H), 2.90 (s, 6H) ppm; ^{13}C NMR (125 MHz, DMSO-d6): δ = 164.31, 151.18, 148.70, 133.61, 129.10, 128.19, 127.81, 117.41, 115.41, 114.85, 112.41, 67.10 ppm.

2-(3-Hydroxyphenyl)-2,3-dihydroquinazolin-4(1H)-one (Table 2, entry 9, ; Fig. S17 and Fig. S18): m.p.: 232–234 °C; ^{1}H NMR (600 MHz, DMSO-d6): δ = 9.49 (s, 1H, O–H), 8.23 (s, 1H, N–H), 7.61 (d, 1H, J=7.68 Hz, Ar–H), 7.24 (t, 1H, J=7.62 Hz, Ar–H), 7.17 (t, 1H, J=7.77 Hz, Ar–H), 7.07 (s, 1H, N–H), 6.91–6.89 (m, 2H, Ar–H), 6.75–6.72 (m, 2H, Ar–H), 6.67 (t, 1H,

J=7.44 Hz, Ar–H), 5.66 (s, 1H, CH) ppm; ^{13}C NMR (150 MHz, DMSO-d6): δ = 163.5, 157.3, 147.8, 143.2, 133.2, 129.3, 127.3, 117.4, 116.9, 115.3, 114.8, 114.3, 113.6, 66.4 ppm.

2-(3-methoxyphenyl)-2,3-dihydroquinazolin-4(1*H*)-one (Table 2, entry 10, ; Fig. S19 and Fig. S20): m.p.: 200–202 °C; ^{1}H NMR (400 MHz, DMSO-d6): *δ* = 8.32 (s, 1H), 7.61 (d, *J* = 7.6 Hz, 1H), 7.31 (t, *J* = 8.0 Hz, 1H), 7.26 (t, *J* = 8.0 Hz, 1H), 7.15 (s, 1H), 7.10-7.06 (m, 2H), 6.92 (d, *J* = 8.4 Hz, 1H), 6.77 (d, *J* = 8.4 Hz, 1H), 6.68 (t, *J* = 7.6 Hz, 1H), 5.73 (s, 1H) , 3.76 (s, 3H) ppm. ^{13}C NMR (100 MHz, DMSO-d6): *δ* = 164.0, 159.7, 148.3, 143.8, 133.8, 129.9, 127.8, 119.4, 117.6, 115.4, 114.9, 114.1, 113.0, 66.7, 55.6 ppm.

2-(*o*-Tolyl)-2,3-dihydroquinazolin-4(1*H*)-one (Table 2, entry 11, ; Fig. S21 and Fig. S22): m.p. 184−186 °C; ^{1}H NMR (400 MHz, $CDCl_3$): *δ* = 7.28 (d, J = 7.6 Hz, 1H), 7.09 (d, J = 7.1 Hz, 1H), 6.76−6.59 (m, 5H), 6.27 (t, J = 8.0 Hz, 1H), 6.14 (d, J = 8.1 Hz, 1H), 5.58 (s, 1H), 5.52 (s, 1H), 4.27 (s, 1H), 1.87 (s, 3H) ppm; ^{13}C NMR (75 MHz, $CDCl_3$): *δ* = 165.4, 147.9, 136.4, 136.1, 134.0, 131.2, 129.5, 128.6, 127.7, 126.6, 119.3, 115.5, 114.78, 65.8, 19.0 ppm.

2-(2-hydroxyphenyl)-2,3-dihydroquinazolin-4(1*H*)-one (Table 2, entry 12; Fig. S23 and Fig. S24): m.p. 197−199 °C; ^{1}H NMR (400 MHz, DMSO-d6): *δ* = 9.86 (s, 1H), 7.94 (s, 1H), 7.63 (dd, J1 = 7.6 Hz, J2 = 1.6 Hz, 1H), 7.35 (dd, J1 = 7.6 Hz, J2 = 1.6 Hz, 1H), 7.22 (dt, J1 = 8.0 Hz, J2 = 1.6 Hz, 1H), 7.15 (dt, J1 = 8.0 Hz, J2 = 1.6 Hz, 1H), 6.87 (dd, J1 = 8.0 Hz, J2 = 1.6 Hz, 1H), 6.81-6.74 (m, 3H), 6.66 (dt, J1 = 7.2 Hz, J2 = 0.8 Hz, 1H), 6.01 (s, 1H) ppm. ^{13}C NMR (100 MHz, DMSO-d6): *δ* = 164.5, 155.1, 148.6, 133.7, 129.8, 127.8, 127.7, 127.6, 119.3, 117.5, 115.8, 115.3, 115.0, 61.7 ppm.

2-(2,4-Dichlorophenyl)-2,3-Dihydroquinazolin-4(1*H*)-One (Table 2, entry 13, Fig. S25 and Fig. S26): m.p. 167-169 °C; ^{1}H NMR (500 MHz, DMSO-d6): *δ* = 8.27 (s, 1H), 7.66 (dd, *J* = 9.4, 4.1 Hz, 3H), 7.50 (dd, *J* = 8.4, 2.1 Hz, 1H), 7.30-7.23 (m, 1H), 7.05 (s, 1H), 6.79-6.69 (m, 2H), 6.12 (s, 1H) ppm; ^{13}C NMR (125 MHz, DMSO-d6): *δ* = 161.89, 145.86, 137.3, 135.26, 132.28, 131.86, 131.24, 128.50, 127.30, 126.01, 125.74, 115.96, 112.95, 61.65 ppm.

2-(4-hydroxy-3-methoxyphenyl)-2,3-dihydroquinazolin-4(1*H*)-one (Table 2, entry 14, ; Fig. S27 and Fig. S28): m.p.: 211–213 °C; ^{1}H NMR (400 MHz, DMSO-d6): *δ* = (ppm) 3.77 (s, 3H, OCH3), 5.67 (s, 1H, benzylic), 6.67–6.71 (t, 1H, J = 7.02 Hz, aromatic), 6.76–6.79 (m., 2H, aromatic), 6.89–6.91 (d.d, 1H, J = 8 Hz, aromatic), 6.98 (s, 1H, –NH), 7.10 (s, 1H, aromatic),

7.23–7.27 (t.d, 1H, J = 8.4 Hz, aromatic), 7.62–7.64 (d, 1H, J = 8 Hz, aromatic), 8.15 (s, 1H, –NH), 9.15 (s, 1H, –OH) ppm. ^{13}C NMR (125 MHz, DMSO-d6): δ = (ppm) 56.04, 67.26, 111.51, 114.91, 115.38, 115.44, 117.63, 120.07, 127.84, 132.39, 133.75, 147.34, 147.88, 148.64, 164.36 ppm.

2-(3,4,5-trimethoxyphenyl)quinazolin-4(3*H*)-one (Table 2, entry 14, ; Fig. S29 and Fig. S30): m.p.: 181–183 °C; ^{1}H NMR (400 MHz, $CDCl_3$): δ = 11.90 (br s, 1H), 8.26 (d, J = 8.0 Hz, 1H), 7.89-7.81 (m, 2H), 7.54-7.50 (m, 3H), 4.05 (s, 6H), 3.96 (s, 3H) ppm. ^{13}C NMR (100 MHz, $CDCl_3$): δ = 153.8 (2-CH), 151.9, 149.2, 141.5, 135.5 (2-CH), 127.8, 127.5, 127.2 (2-CH), 126.3, 105.1 (2-CH), 61.2, 56.6 (2C-OMe) ppm.

2. ^{1}H and ^{13}C NMR spectra of compounds

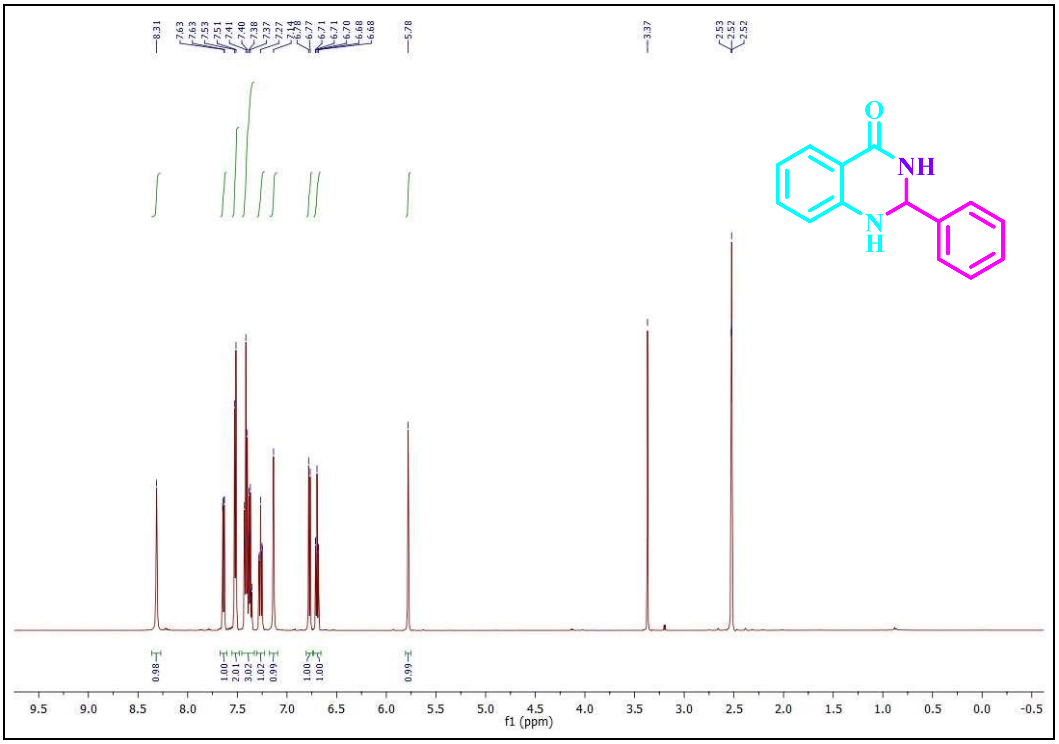

Fig. S1. ^{1}H NMR of 2-phenyl-2,3-dihydroquinazolin-4(1*H*)-one.

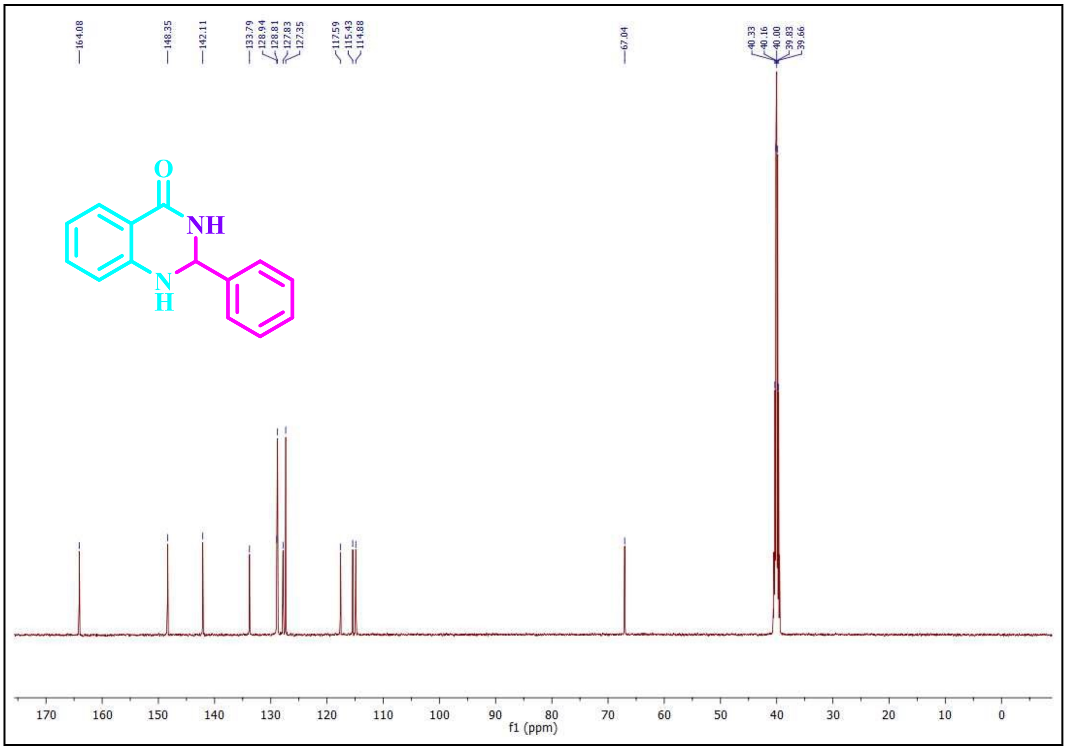

Fig. S2. ^{13}C NMR of 2-phenyl-2,3-dihydroquinazolin-4(1*H*)-one.

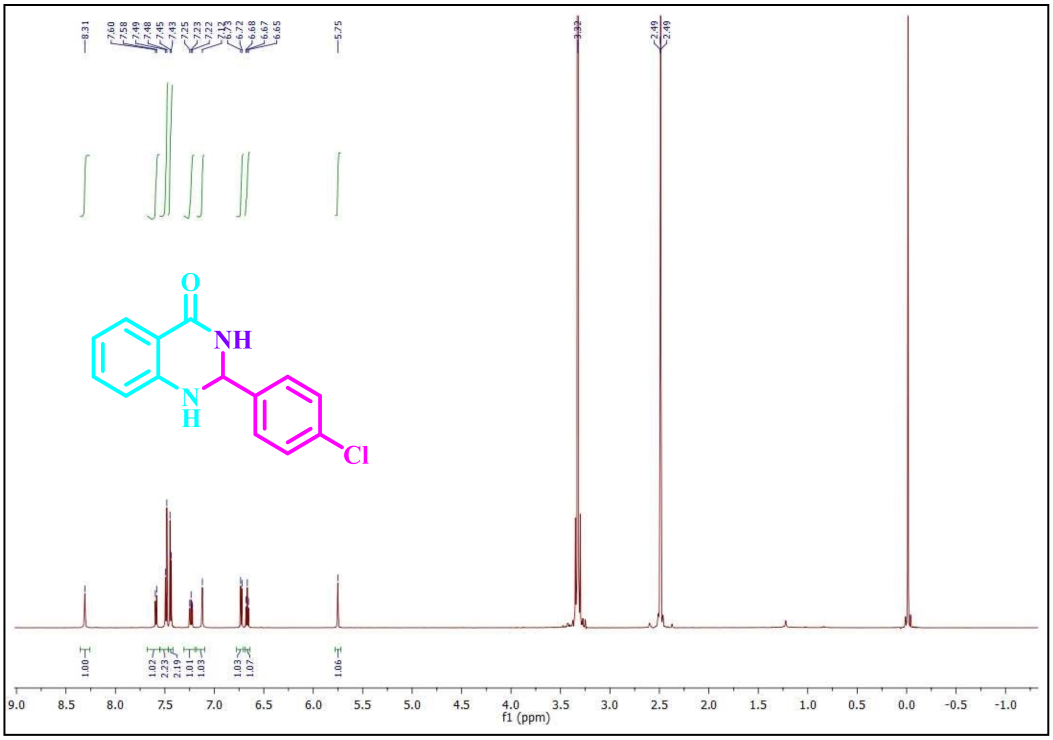

Fig. S3. ^{1}H NMR of 2-(4-chlorophenyl)-2,3-dihydroquinazolin-4(1*H*)-one.

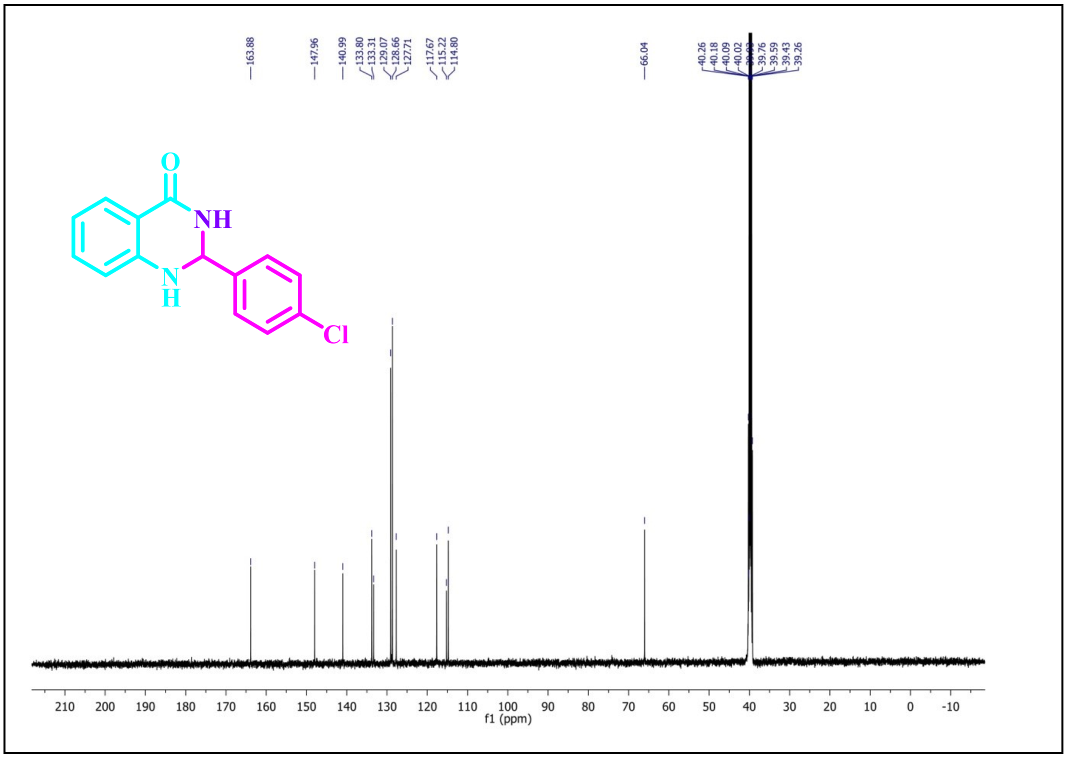

Fig. S4. ^{13}C NMR of 2-(4-chlorophenyl)-2,3-dihydroquinazolin-4(1*H*)-one.

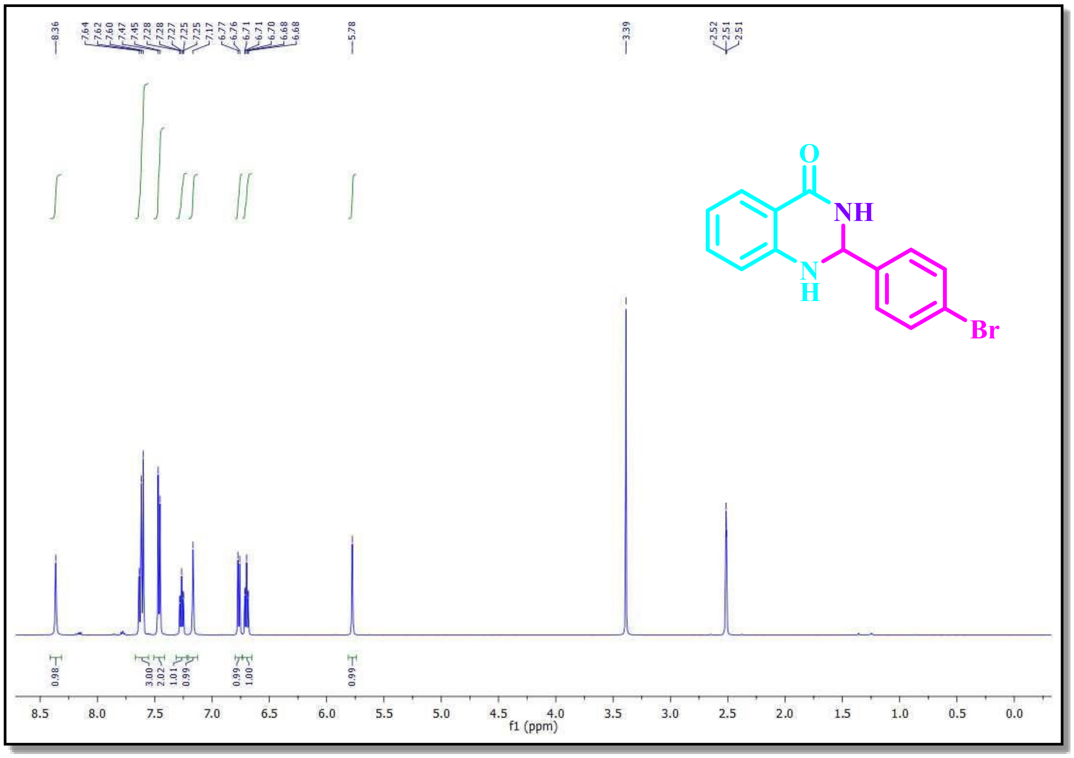

Fig. S5. ^{1}H NMR of 2-(4-bromophenyl)-2,3-dihydroquinazolin-4(1*H*)-one.

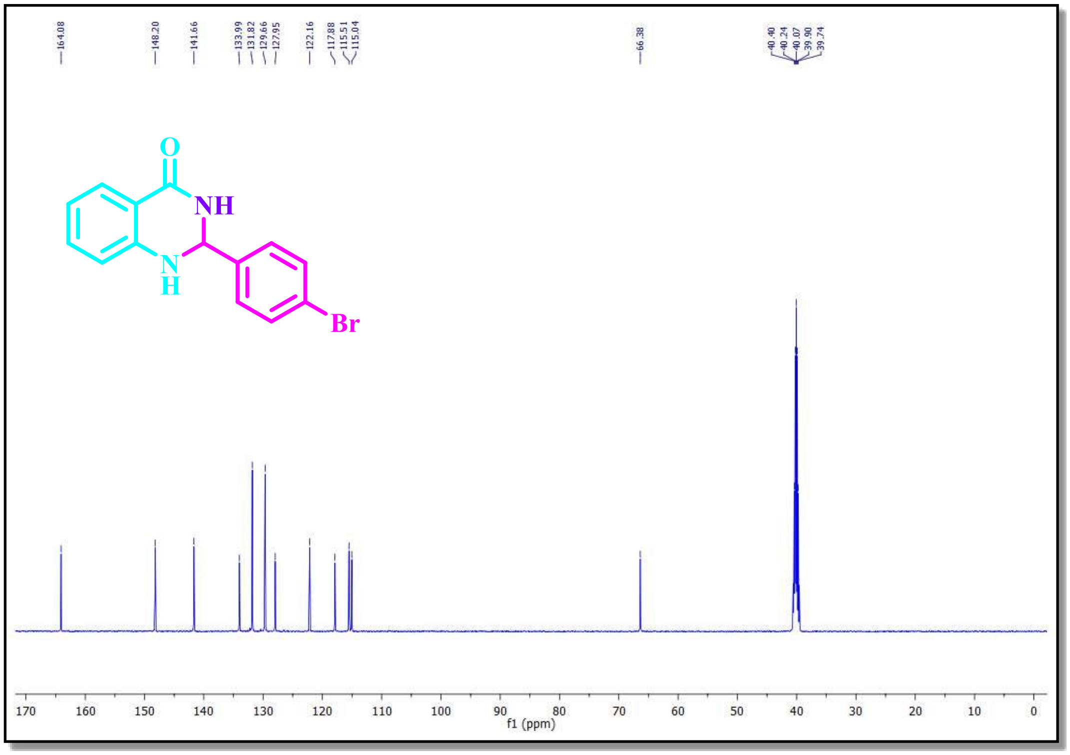

Fig. S6. ^{13}C NMR of 2-(4-bromophenyl)-2,3-dihydroquinazolin-4(1*H*)-one.

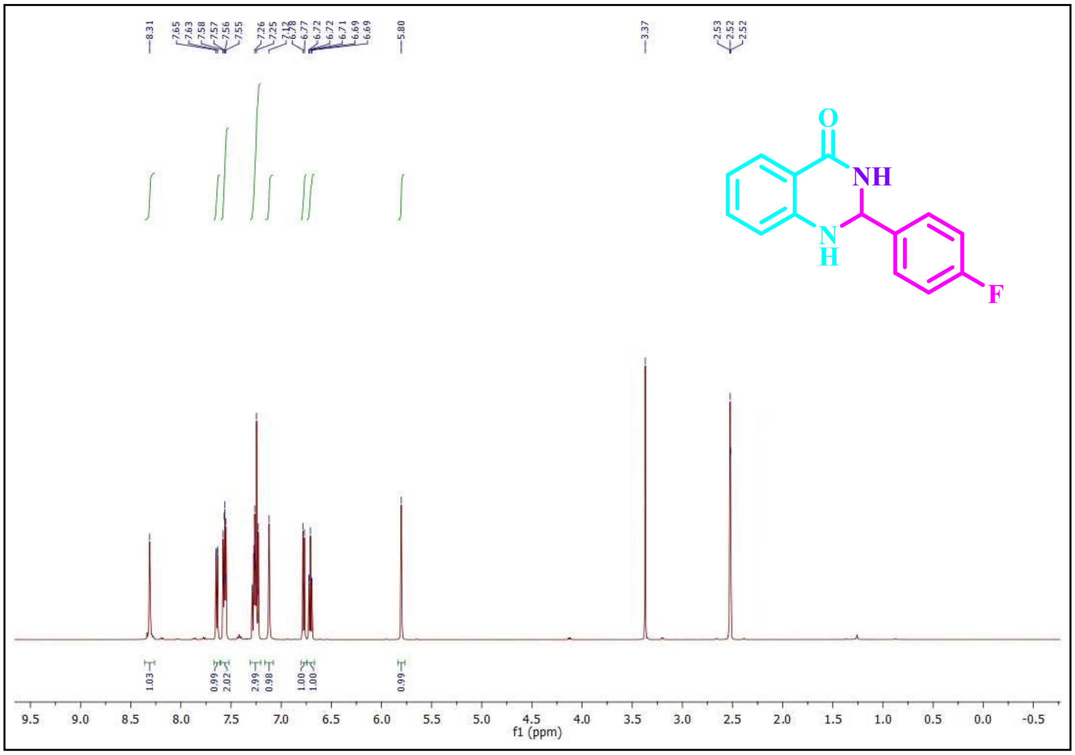

Fig. S7. ^{1}H NMR of 2-(4-flourophenyl)-2,3-dihydroquinazolin-4(1*H*)-one.

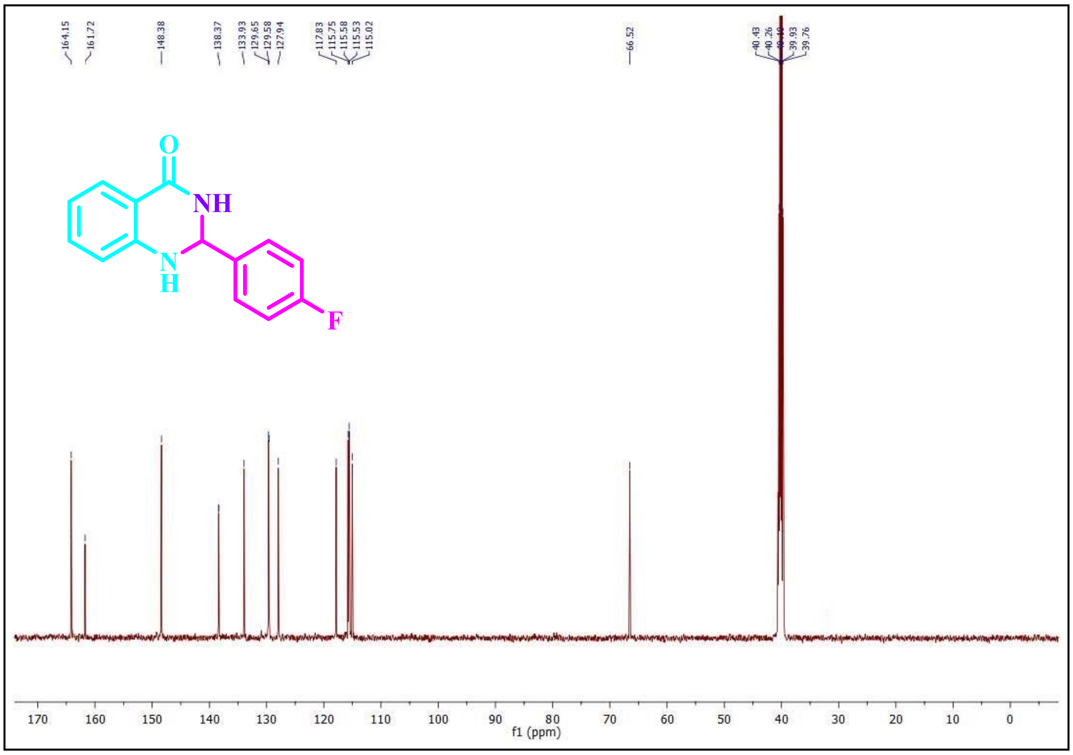

Fig. S8. ^{13}C NMR of 2-(4-flourophenyl)-2,3-dihydroquinazolin-4(1*H*)-one.

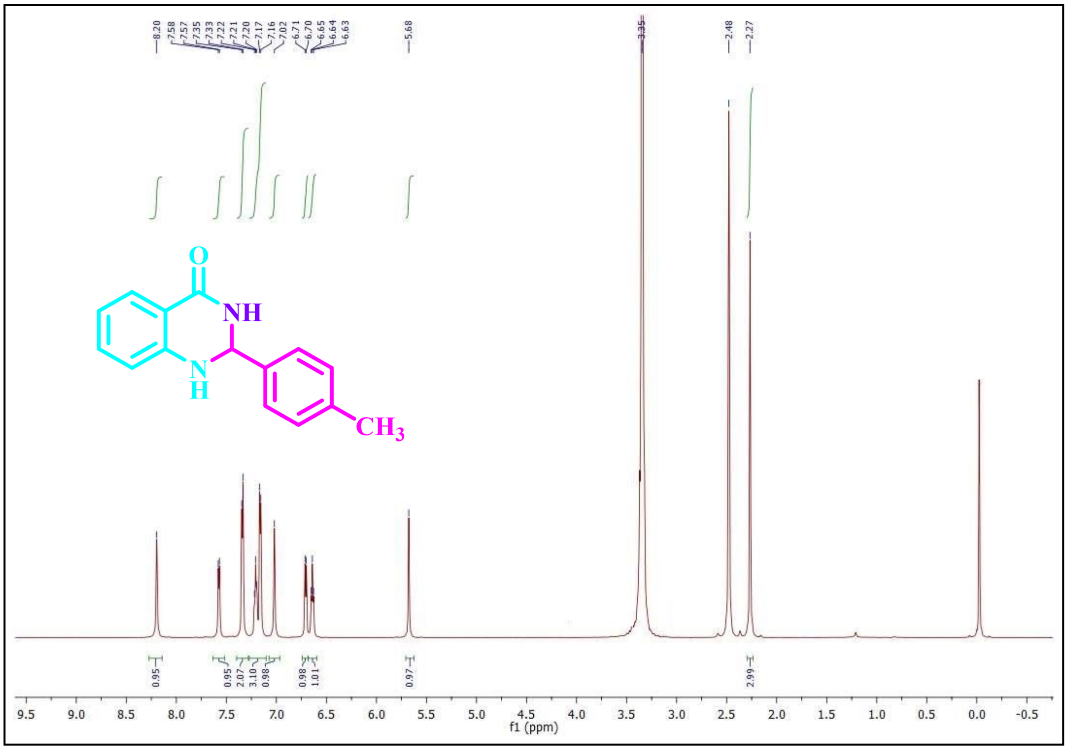

Fig. S9. ^{1}H NMR of 2-(*p*-tolyl)-2,3-dihydroquinazolin-4(1*H*)-one.

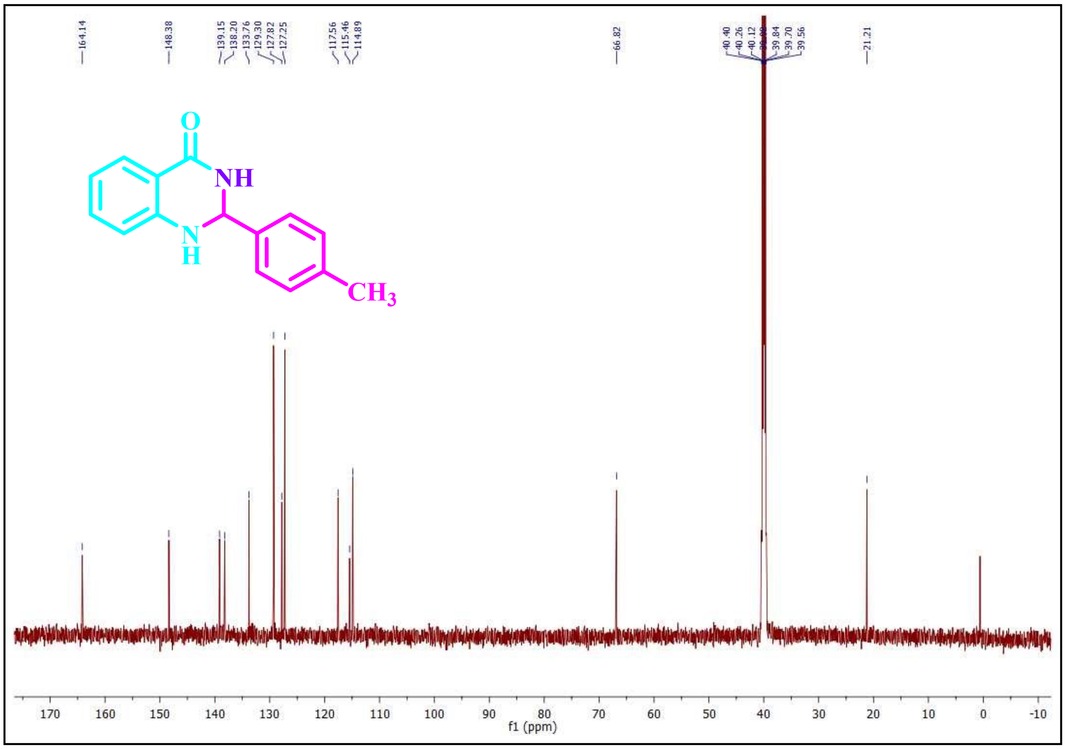

Fig. S10. ^{13}C NMR of 2-(*p*-tolyl)-2,3-dihydroquinazolin-4(1*H*)-one.

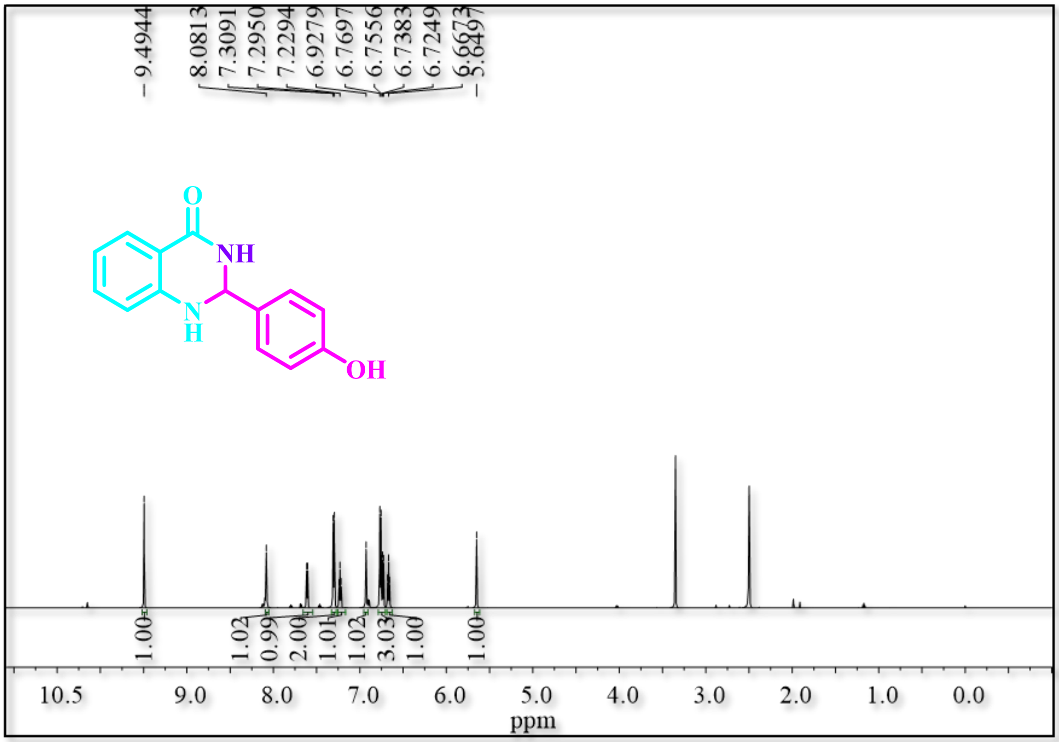

Fig. S11. ^{1}H NMR of 2-(4-Hydroxy)-2, 3-dihydroquinazolin-4(1*H*)-one.

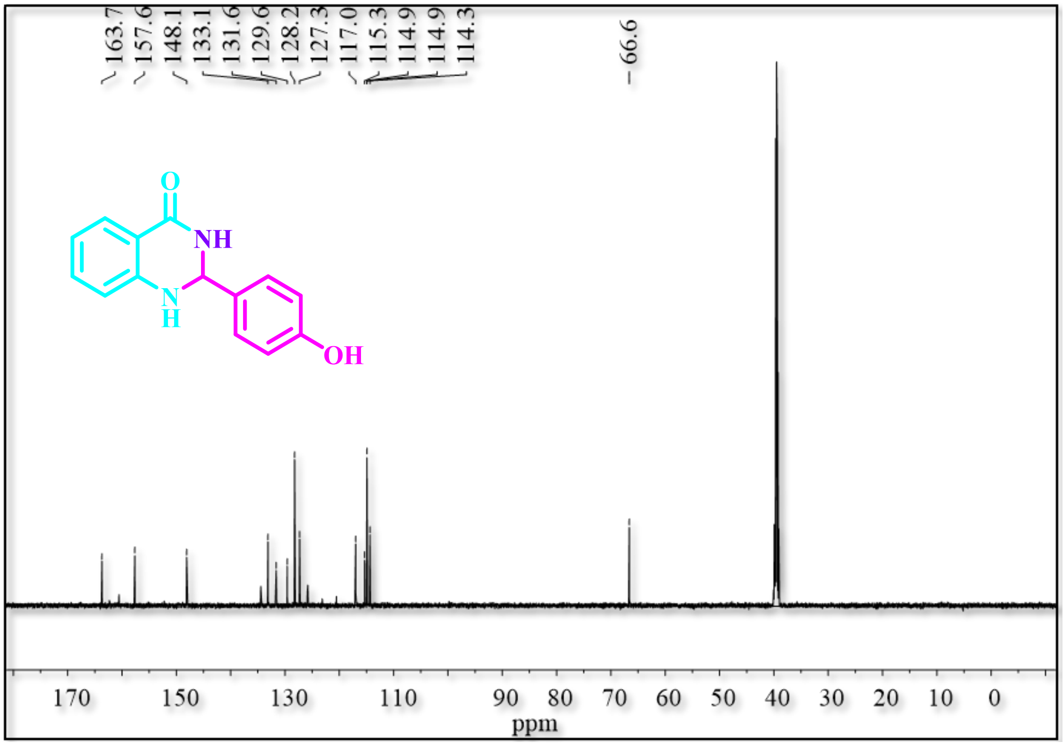

Fig. S12. ^{13}C NMR of 2-(4-Hydroxy)-2, 3-dihydroquinazolin-4(1*H*)-one.

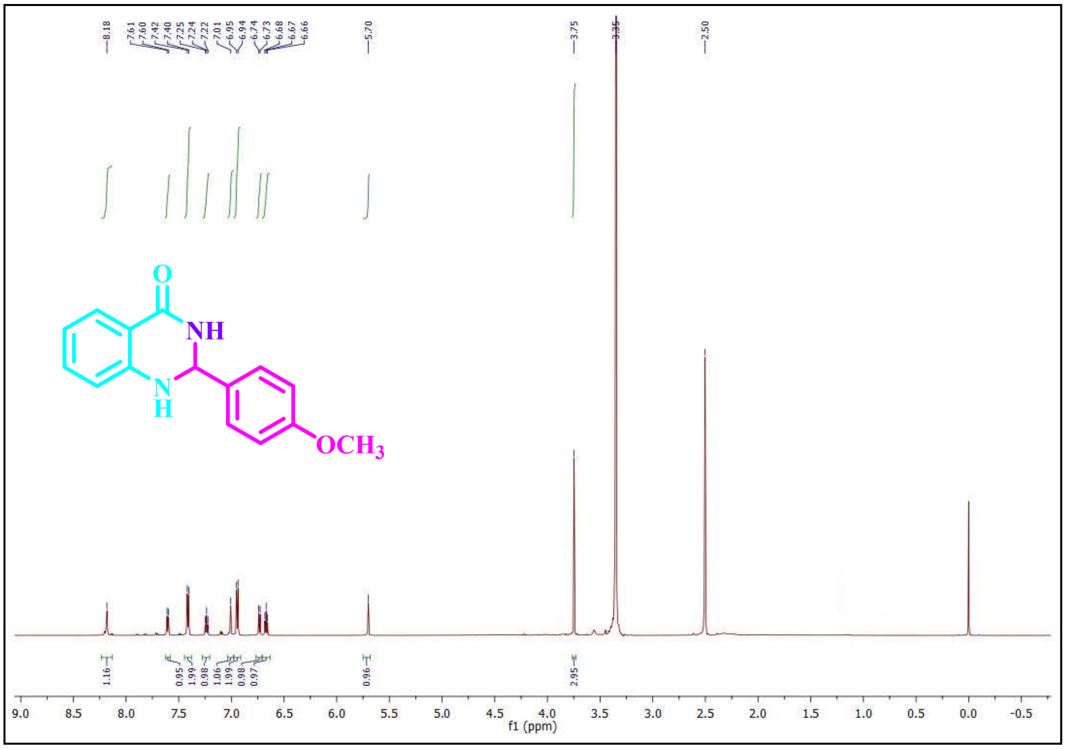

Fig. S13. ^{1}H NMR of 2-(4-Methoxyphenyl)-2, 3-dihydroquinazolin-4(1*H*)-one.

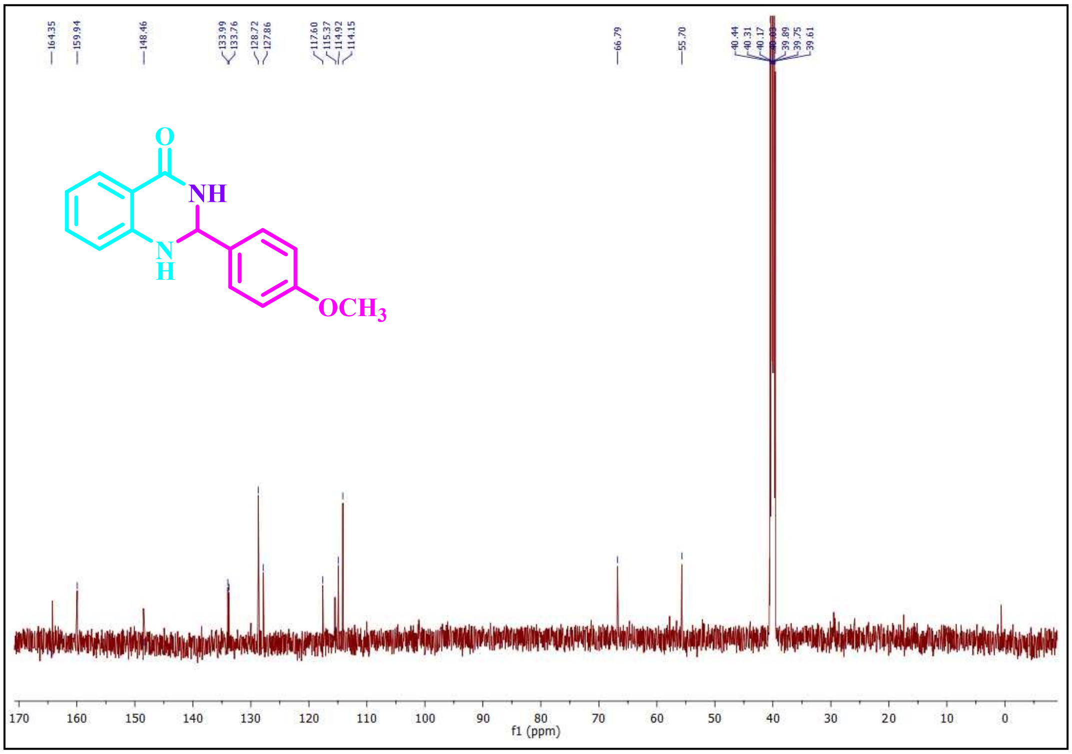

Fig. S14. ^{13}C NMR of 2-(4-Methoxyphenyl)-2, 3-dihydroquinazolin-4(1*H*)-one.

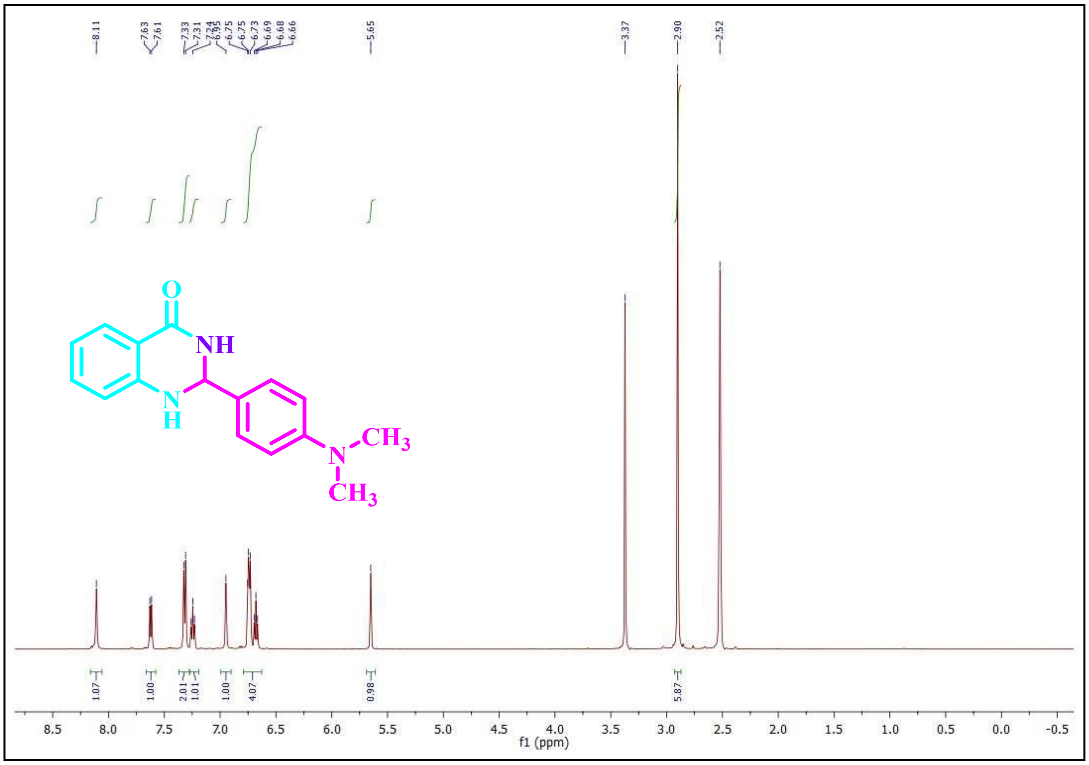

Fig. S15. ^{1}H NMR of 2-(4-(dimethylamino)phenyl)-2,3-dihydroquinazolin-4(1*H*)-one.

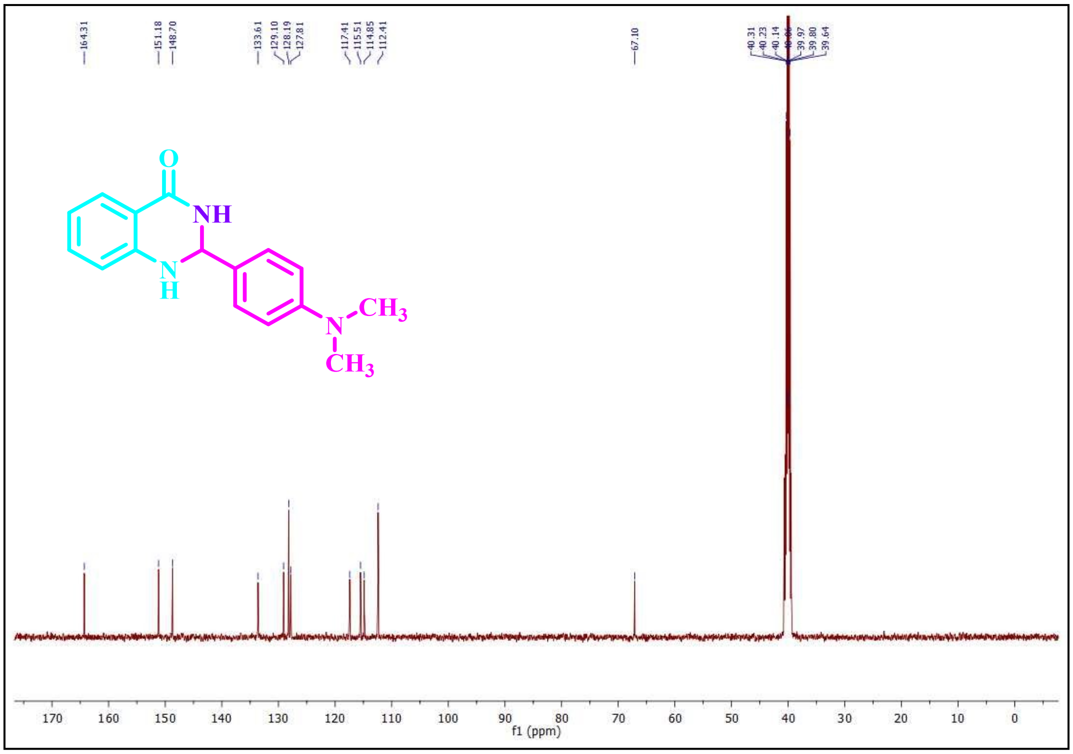

Fig. S16. ^{13}C NMR of 2-(4-(dimethylamino)phenyl)-2,3-dihydroquinazolin-4(1*H*)-one.

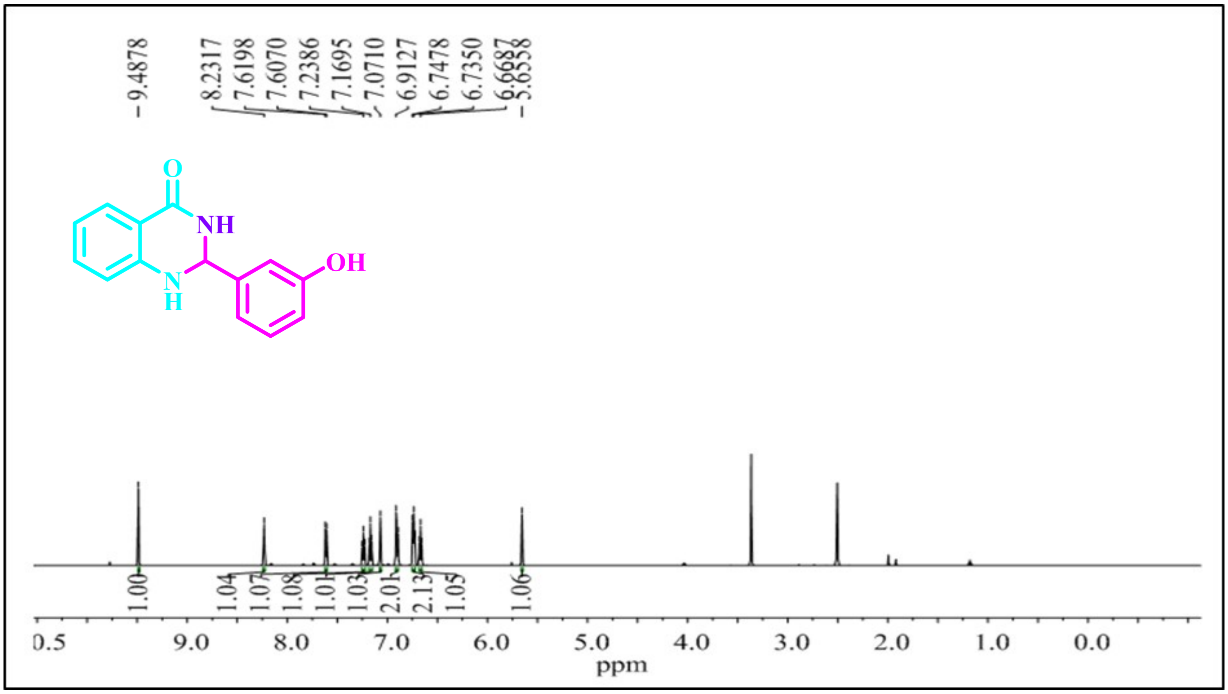

Fig. S17. ^{1}H NMR of 2-(3-hydroxyphenyl)-2,3-dihydroquinazolin-4(1*H*)-one.

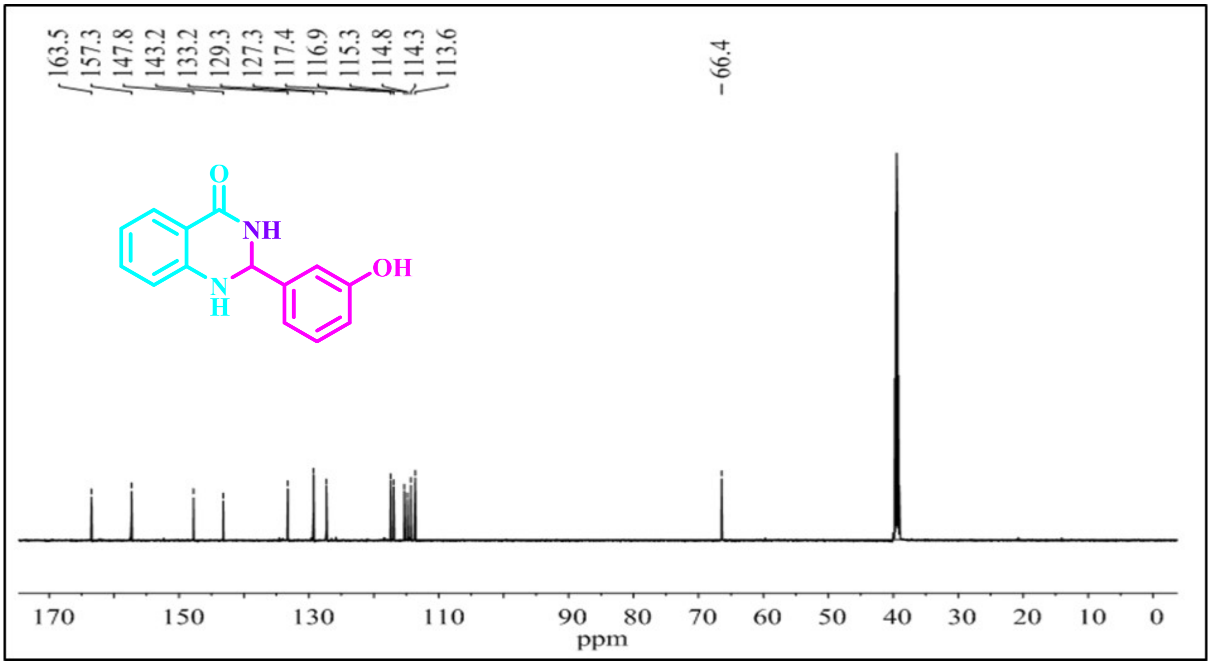

Fig. S18. ^{13}C NMR of 2-(3-hydroxyphenyl)-2,3-dihydroquinazolin-4(1*H*)-one.

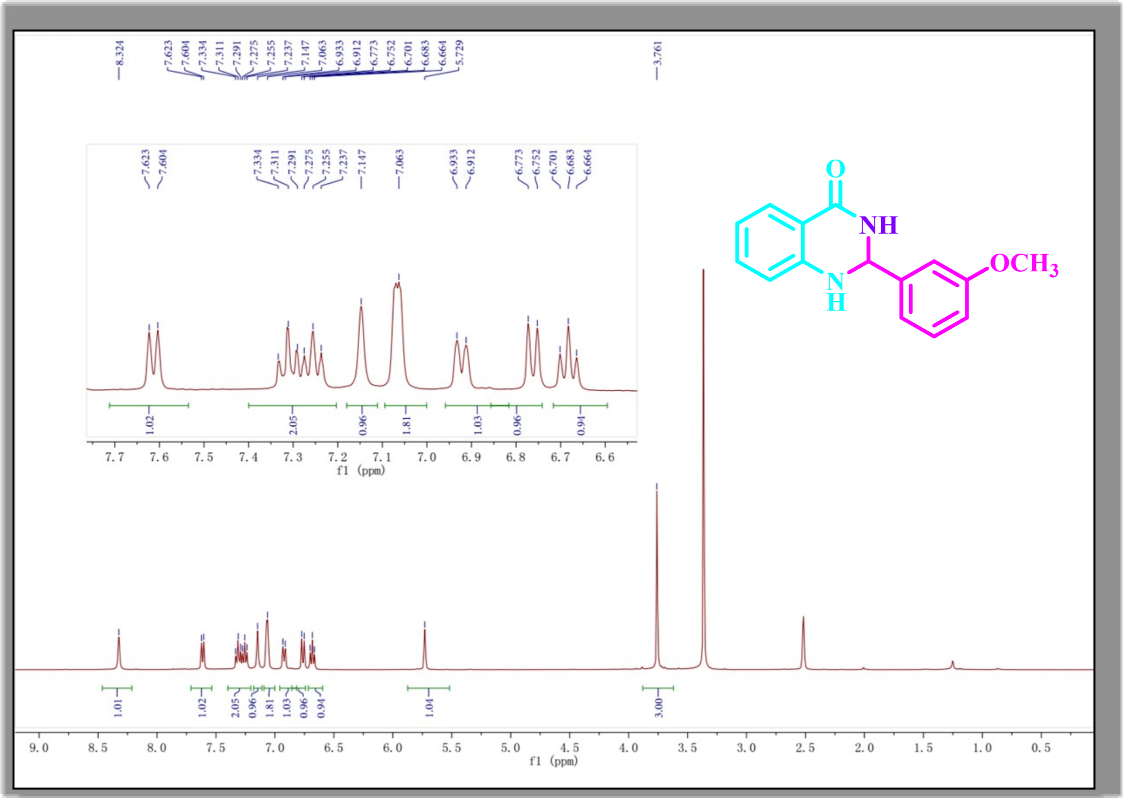

Fig. S19. ^{1}H NMR of 2-(3-methoxyphenyl)-2,3-dihydroquinazolin-4(1*H*)-one.

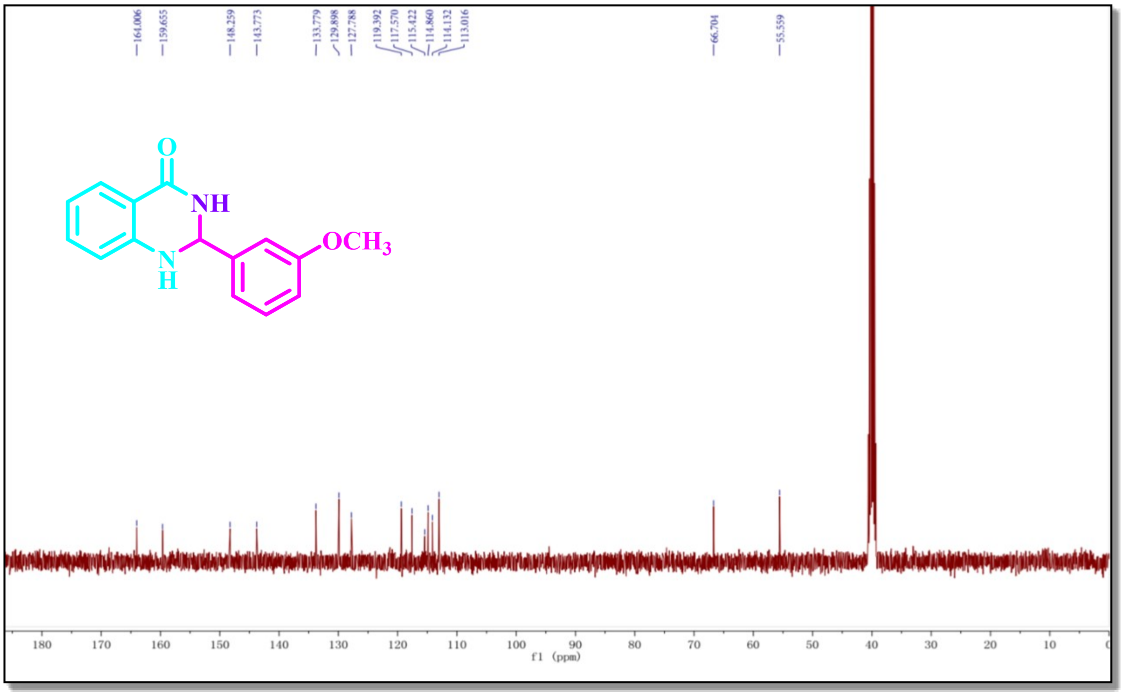

Fig. S20. ^{13}C NMR 2-(3-methoxyphenyl)-2,3-dihydroquinazolin-4(1*H*)-one.

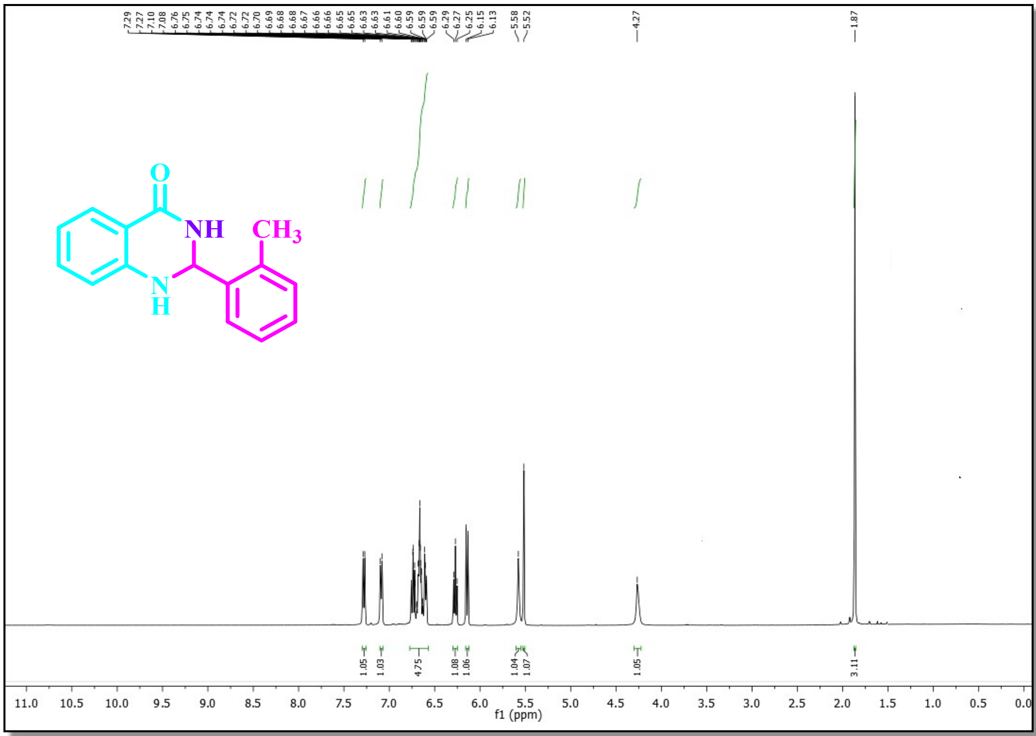

Fig. S21. ^{1}H NMR of 2-(*o*-tolyl)-2,3-dihydroquinazolin-4(1*H*)-one.

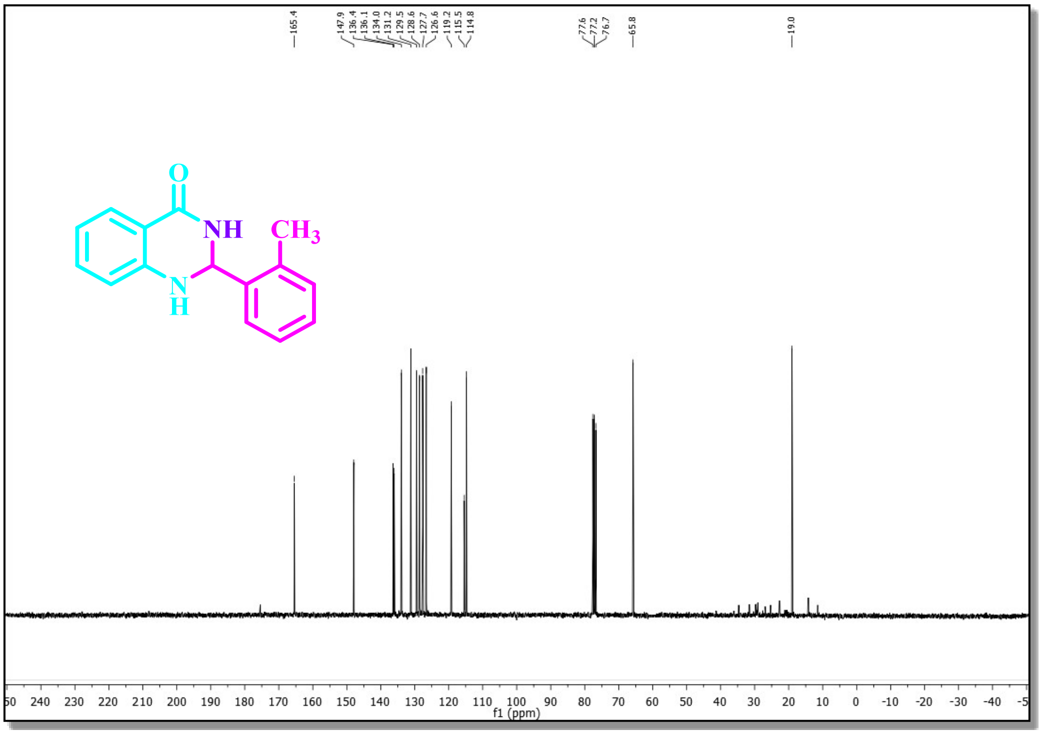

Fig. S22. ^{13}C NMR of 2-(*o*-tolyl)-2,3-dihydroquinazolin-4(1*H*)-one.

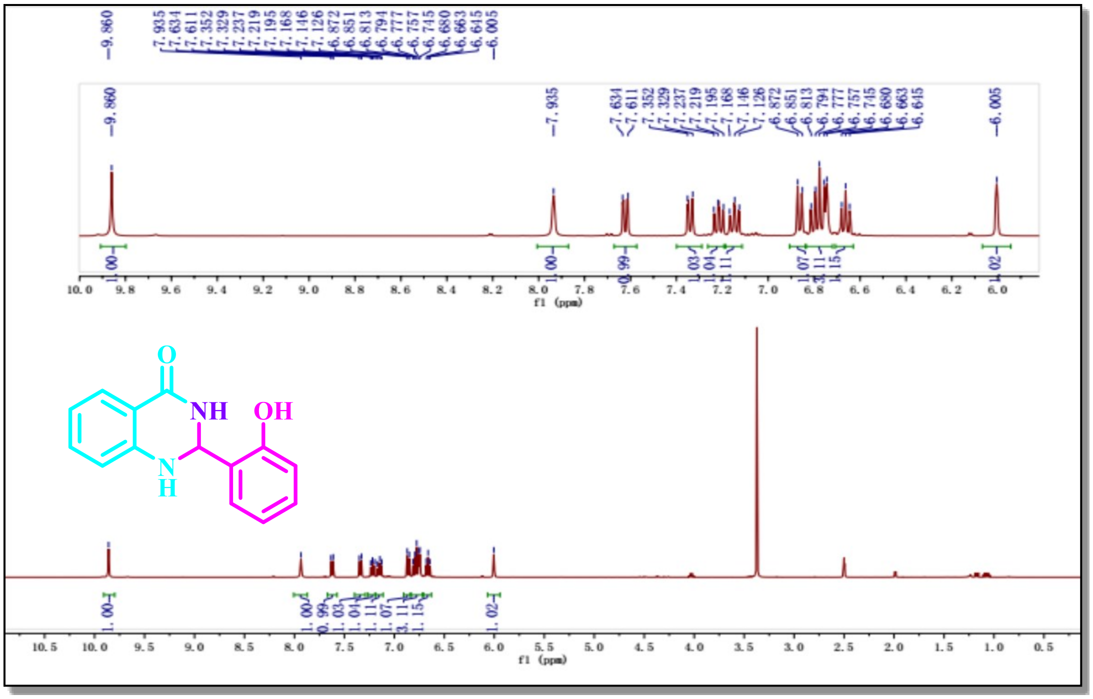

Fig. S23. ^{1}HNMR of 2-(2-hydroxyphenyl)-2,3-dihydroquinazolin-4(1*H*)-one.

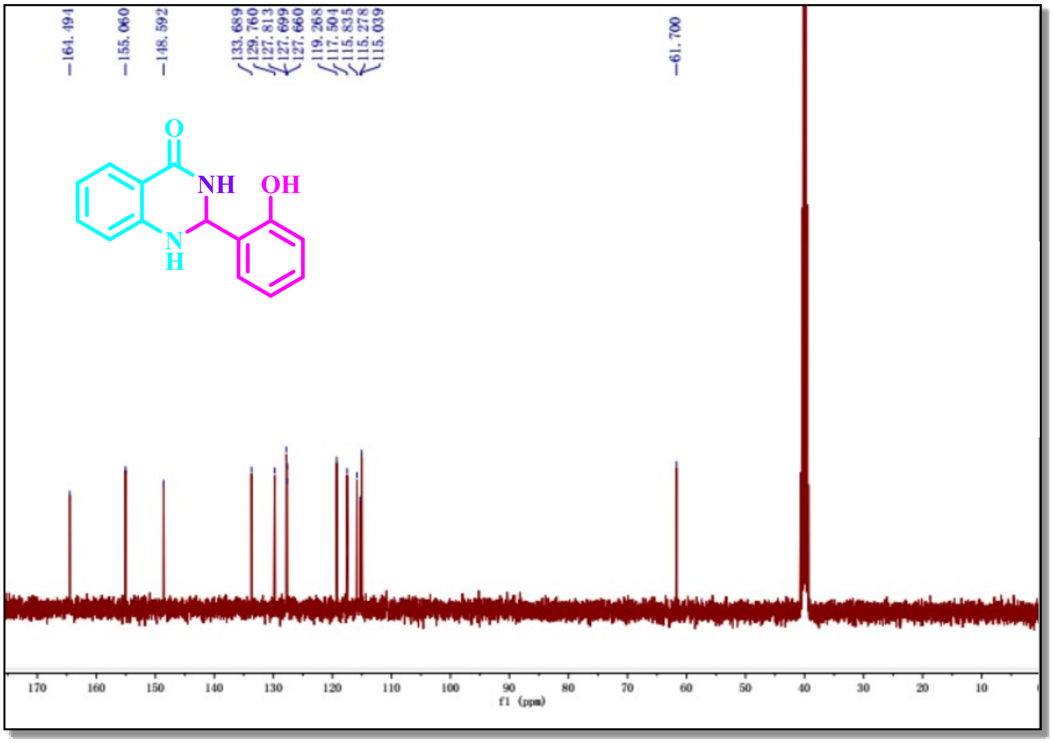

Fig. S24. ^{13}CNMR of 2-(2-hydroxyphenyl)-2,3-dihydroquinazolin-4(1*H*)-one.

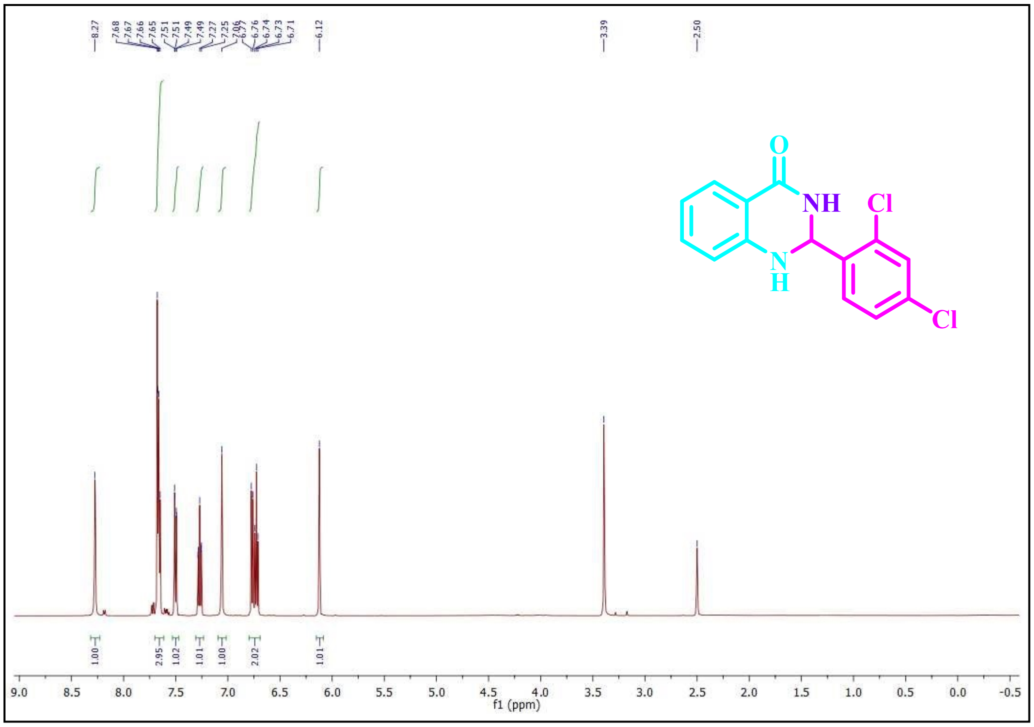

Fig. S25. ^{1}H NMR spectrum of 2-(2,4-dichlorophenyl)-2,3 dihydroquinazolin-4(1*H*)-one.

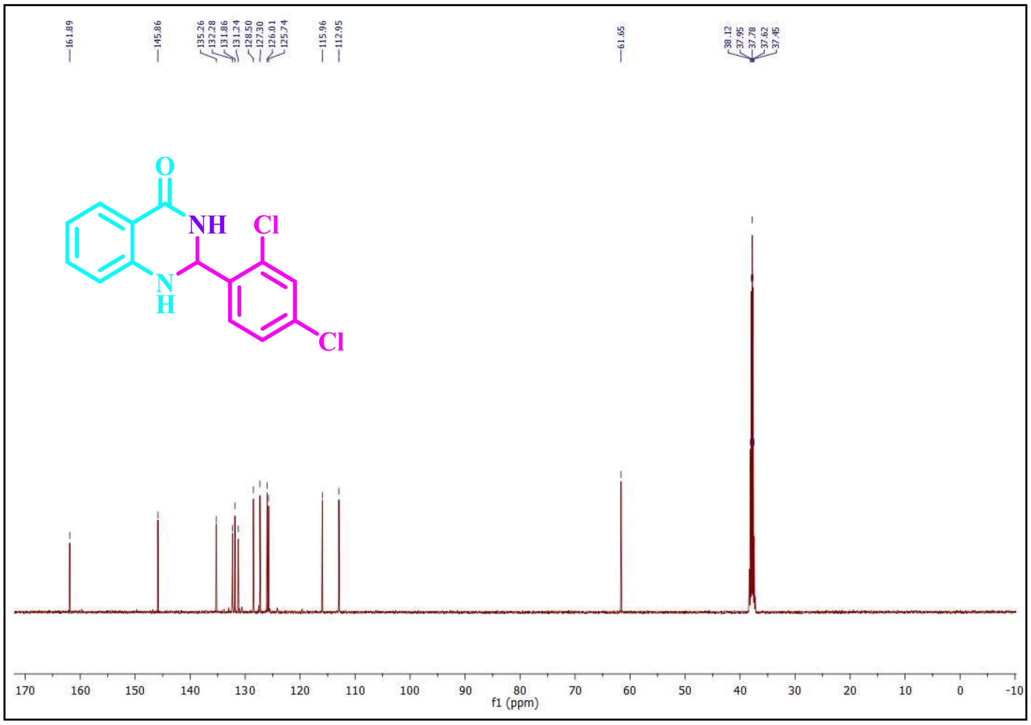

Fig. S26. ^{13}C NMR spectrum of 2-(2,4-dichlorophenyl)-2,3-dihydroquinazolin-4(1*H*)-one.

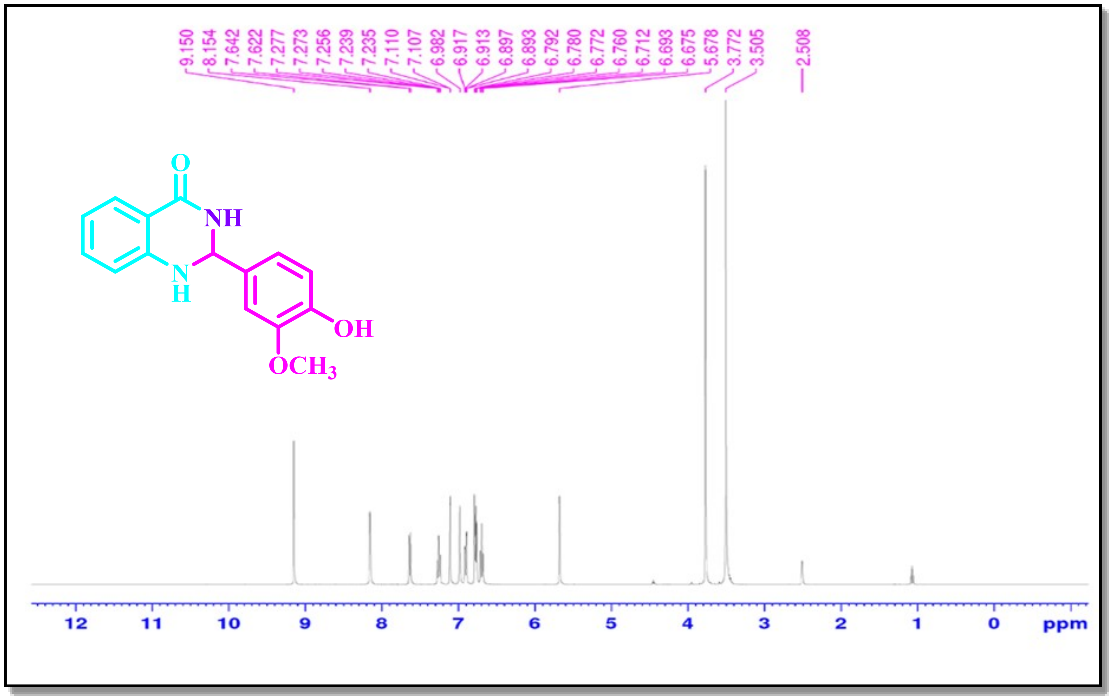

Fig. S27. ^{1}H NMR of 2-(4-hydroxy-3-methoxyphenyl)-2,3-dihydroquinazolin-4(1*H*)-one.

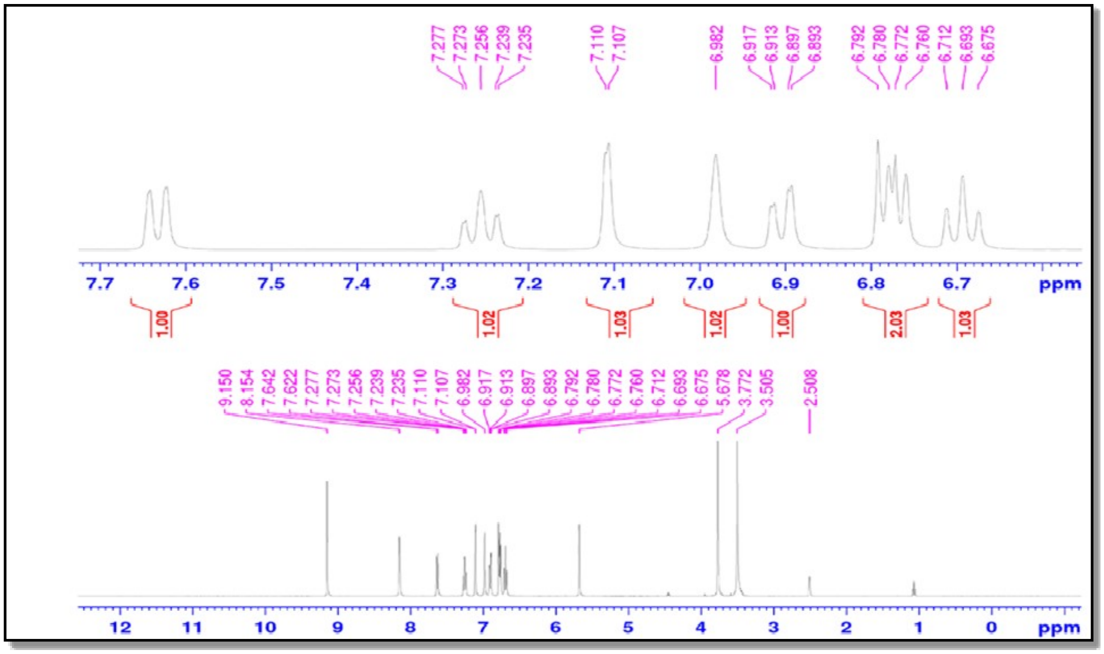

Fig. S28. ^{13}C NMR of 2-(4-hydroxy-3-methoxyphenyl)-2,3-dihydroquinazolin-4(1*H*)-one.

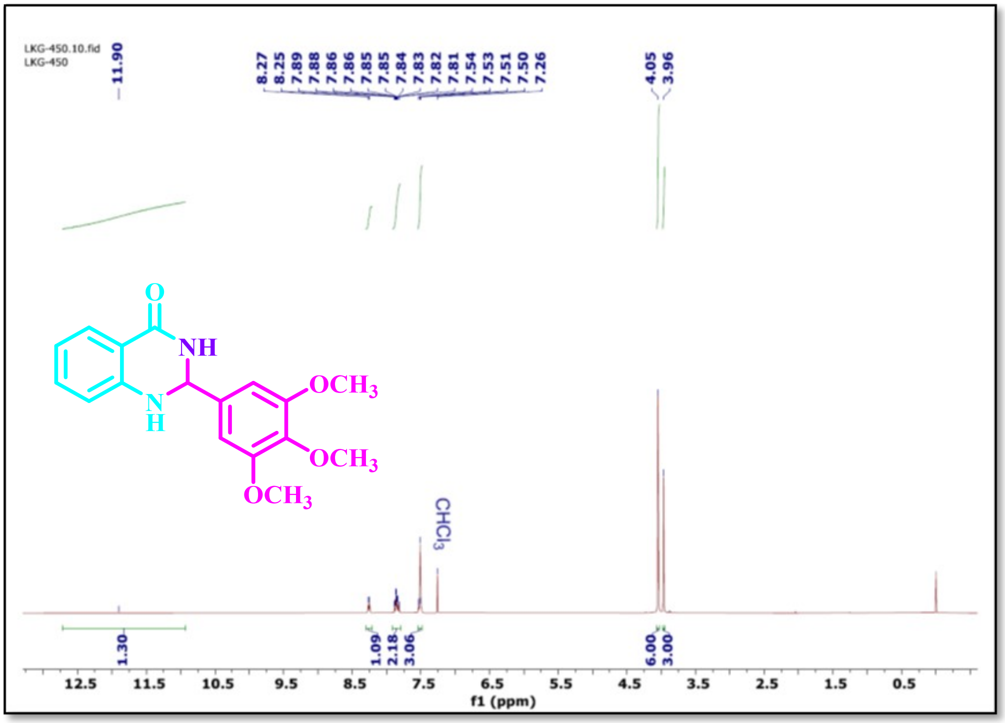

Fig. S29. ^{1}H NMR of (3,4,5-trimethoxyphenyl)-2,3-dihydroquinazolin-4(1*H*)-one.

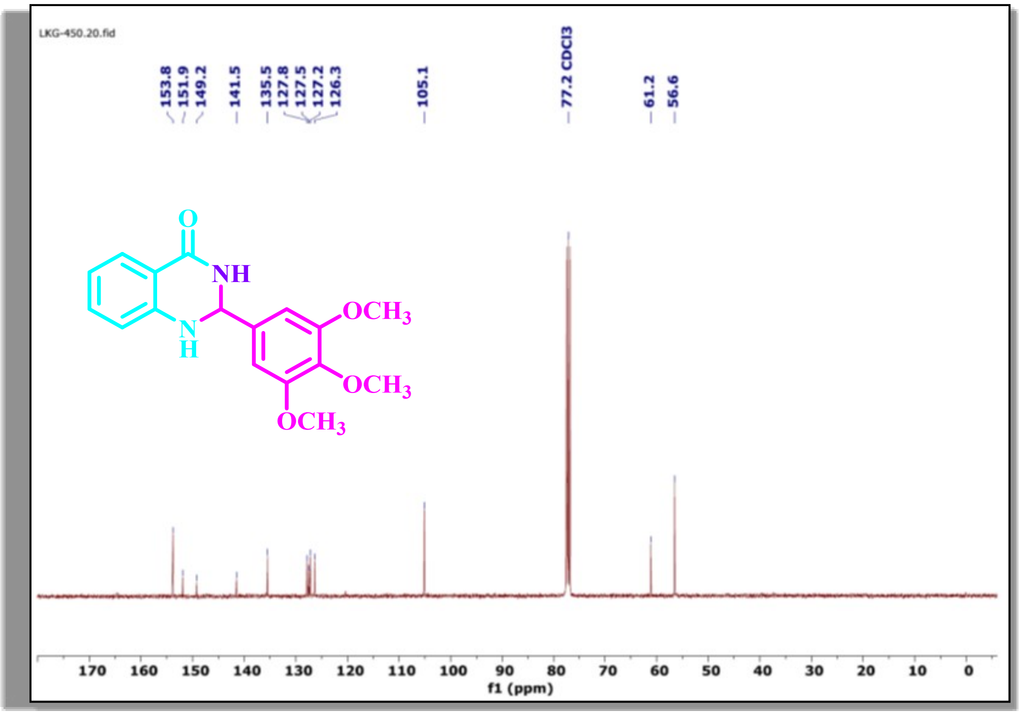

Fig. S30. ^{13}C NMR of 2-(3,4,5-trimethoxyphenyl)-2,3-dihydroquinazolin-4(1*H*)-one.
